# Exploring the potential applications of lead-free organic–inorganic perovskite type [NH_3_(CH_2_)_*n*_NH_3_]*M*Cl_4_ (*n* = 2, 3, 4, 5, and 6; *M* = Mn, Co, Cu, Zn, and Cd) crystals

**DOI:** 10.1038/s41598-024-62705-9

**Published:** 2024-05-23

**Authors:** Ae Ran Lim

**Affiliations:** 1 Graduate School of Carbon Convergence Engineering, Jeonju University, Jeonju, 55069 South Korea; 2 Department of Science Education, Jeonju University, Jeonju, 55069 South Korea

**Keywords:** Materials science, Physics

## Abstract

The organic–inorganic hybrid perovskite compounds have been extensively studied since the dawn of a new era in the field of photovoltaic applications. Up to now, perovskites have proven to be the most promising in terms of power conversion efficiency; however, their main disadvantages for use in solar cells are toxicity and chemical instability. Therefore, it is essential to develop a hybrid perovskite that can be replaced with lead-free materials. This review focuses on the possibility of applying lead-free organic–inorganic perovskite types [NH_3_(CH_2_)_*n*_NH_3_]*M*Cl_4_ (*n* = 2, 3, 4, 5, and 6; *M* = Mn, Co, Cu, Zn, and Cd) crystals. We are seeking organic–inorganic hybrid perovskite materials with very high temperature stability or without phase transition temperature, and thermal stability. Thus, by considering the characteristics according to the methylene lengths and the various transition metals, we aim to identify improved materials meeting the criteria mentioned above. Consequently, the physicochemical properties of organic–inorganic hybrid perovskite [NH_3_(CH_2_)_*n*_NH_3_]*M*Cl_4_ regarding the effects of various transition metal ions of the anion and the methylene lengths of the cation are expected to promote the development and application of lead-free hybrid perovskite solar cells.

Recent research has focused on exploring new and improved functional materials within the organic–inorganic hybrid perovskite materials. The wide application scope and rapid development speed of various studies on organic–inorganic hybrid compounds have garnered significant attention^1–18^. The optical properties and structural flexibility of these compounds are determined by organic cations, while their thermal and mechanical properties are governed by inorganic anions^19^. Physicochemical characteristics are influenced by factors such as the properties of organic cations and the coordination geometry of inorganic metal halide anions constituting the crystal^6,7,18,20–25^. These materials are gaining prominence for their unique ability to create diverse and excellent materials by selectively leveraging the advantages of both organic and inorganic components. Furthermore, ferroelasticity is commonly observed in compounds with perovskite crystal structures, and the ferroelastic twin domains in organic–inorganic hybrid perovskites are drawing much attention^26,27^. The development of ferroelastic semiconductors associated with hybrid perovskite fabrication presents significant challenges^7^. Moreover, the successful integration of ferroelectric properties in hybrid perovskites positions them ideally for potential applications in flexible wearable devices^28,29^.

Initially, thin-film photovoltaic devices based on organic–inorganic hybrid CH_3_NH_3_Pb*X*_3_ (*X* = Cl, Br, and I) were utilized as solar cells. The power conversion efficiency of lead halide perovskite CH_3_NH_3_Pb*X*_3_ has enhanced from 3.8% to more than 20%. Despite advancements in employing 3-dimensional (3D) CH_3_NH_3_Pb*X*_3_ as hybrid solar cells, these perovskites are highly susceptible to humid air and are toxic due to the presence of Pb, necessitating their replacement with environmentally friendly hybrid perovskite solar cells^26,27,30–35^. The primary drawbacks for their use in solar cells are the toxicity and chemical instability of halide perovskites. A lead-free hybrid perovskite based on methylammonium tin iodide was developed and showed initial efficiencies of up to 6.4%. And, the band gap energy relevant for photovoltaic applications of the 2D perovskite hybrid was found to be 1.75–2.65 eV. In terms of stability and safety, Bi alkyl ammonium has also been reported as a promising example of a lead-free and eco-friendly hybrid perovskite material for solar cell applications^3,8^. Additionally, novel groups of perovskite materials, such as [(CH_3_)_2_NH_2_]Zn(HCOO)_3_, composed of an organic cation and a metal ion, have been discussed^36–42^. These materials show potential for application in memory manipulation devices and next-generation memory storage technology.

Lead-free organic–inorganic hybrid compounds, [NH_3_(CH_2_)_*n*_NH_3_]*MX*_4_ (*n* = 2, 3, 4, …; *M* = transition metal (Mn, Co, Cu, Zn, and Cd); *X* = halogen ion (Cl, Br, I)), with one-dimensional (1D) and two-dimensional (2D) structures, has emerged^1–9,23,24,34,43–47^. The toxicity of heavy metals such as Co and Cd has long been known but accidental exposures of large populations to these elements remain unfortunately a topical issue^48–52^. These compounds consist of organic [NH_3_(CH_2_)_*n*_NH_3_] cations located between inorganic anions along the longest axis of the single crystal. Recently, research on [NH_3_(CH_2_)_*n*_NH_3_]*MX*_2_*X’*_2_, composed of different halogen ions, was conducted by Abdel-Aal et al.^53–55^. The physical and chemical properties of the organic–inorganic hybrid perovskite compounds depend on the characteristics of the organic cations and the coordination geometry of the inorganic anions; (*MX*_4_)^2−^ or (*MX*_6_)^2−^^6,18,24,56–62^. When the transition metal *M* = Mn, Cu, or Cd, the structure consists of alternating corner-shared octahedral (*MX*_6_)^2−^ units (as exemplified by the [NH_3_(CH_2_)_5_NH_3_]MnCl_4_ crystal shown in Fig. 1a). The organic and inorganic layers form infinite 2D structures, connected by N − H∙∙∙Cl hydrogen bonds. In the case of *M* = Co or Zn, the structure comprises tetrahedral (*MX*_4_)^2−^ units positioned between layers of organic cations (as exemplified by the [NH_3_(CH_2_)_6_NH_3_]ZnCl_4_ crystal shown in Fig. 1b). The organic and inorganic layers form infinite 1D structures, connected by N − H∙∙∙Cl hydrogen bonds. The NH_3_ ions bonded at both ends of the organic chain are consistent with the halide ions in the inorganic layer. When the methylene length in the cation is greater than 4, structural rearrangement due to conformational changes in the chains becomes an important factor^63^. The distance between two inorganic layers varies greatly depending on the length of the organic chain, and, in particular, structural rearrangement and geometry due to changes in the length of the cation are also very important factors.

**Figure 1:**
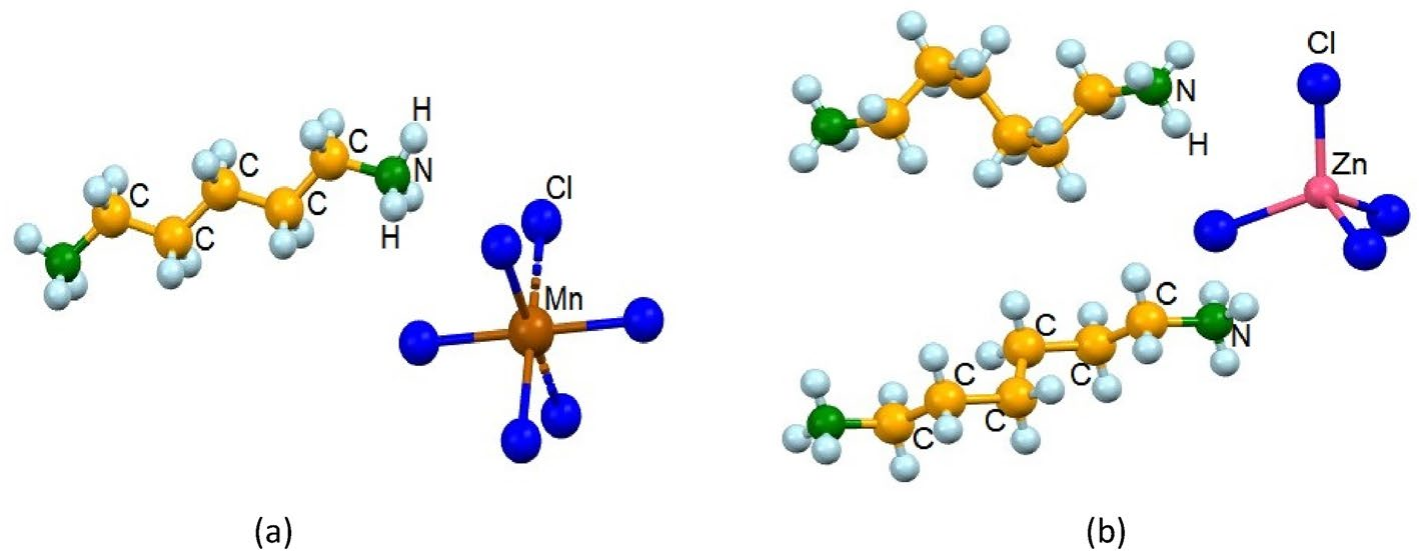
The atomic numbering scheme of (**a**) [NH_3_(CH_2_)_5_NH_3_] cation and MnCl_6_ anion in [NH_3_(CH_2_)_5_NH_3_]MnCl_4_ at 300 K and (**b**) [NH_3_(CH_2_)_6_NH_3_] cation and ZnCl_4_ anion in [NH_3_(CH_2_)_6_NH_3_]ZnCl_4_ at 300 K.

Previous studies on [NH_3_(CH_2_)_*n*_NH_3_]*M*Cl_4_ (*n* = 2, 3, 4, 5, and 6; *M* = Mn, Co, Cu, Zn, and Cd) single crystals have been reported as follows: the research related to [NH_3_(CH_2_)_*n*_NH_3_]MnCl_4_ with *n* values of 2, 3, 4, and 5, and *M* = Mn, was discussed primarily in research reports focusing on the phase transition temperature (T_C_) and crystal structures^63–68^. Their crystallographic characteristics, dielectric properties, and photoluminescence properties were reported^9,69–71^. For *M* = Co, previous studies by Abdel-Aal et al.^72^ and Criado et al.^73^ determined the crystal structures for [NH_3_(CH_2_)_3_NH_3_]CoCl_4_ and [NH_3_(CH_2_)_5_NH_3_]CoCl_4_, respectively. Additionally, Abdel-Aal et al.^62^ discussed the synthesis, structure, and lattice energy of the hybrid organic–inorganic perovskite [NH_3_(CH_2_)_4_NH_3_]CoCl_4_. Regarding *M* = Cu, investigations into the structure, magnetic, and optical properties of the layer-type [NH_3_(CH_2_)_*n*_NH_3_]CuCl_4_ (*n* = 2, 3, 4, and 5) were conducted^23,25,64,74–79^. Iqbal et al.^80^ reported a study on Raman scattering at temperatures above and below the magnetic ordering temperature of 149 K. To date, the structures of [NH_3_(CH_2_)_*n*_NH_3_]ZnCl_4_ (*n* = 2, 3, 4, 5, and 6) have been reported by X-ray diffraction analysis^60,81–83^. Finally, in the case of *M* = Cd, several studies on [NH_3_(CH_2_)_*n*_NH_3_]CdCl_4_ crystals have been reported in the past^84–90^, focusing on structural, thermal, and vibrational properties. Kind et al.^63^ discussed the phase transitions of [NH_3_(CH_2_)_5_NH_3_]CdCl_4_ using ^35^Cl nuclear magnetic resonance (NMR), dilatation and birefringence measurements, and domain investigations. In particular, the electric and optical characteristics of [NH_3_(CH_2_)_*n*_NH_3_]*M*Cl_4_ perovskites, as these properties are crucial for solar cell applications, have been discussed by several researchers^22,23,25,44,63,71,91–93^. However, better optoelectronic research on lead-free metal halide materials for photovoltaic applications is still in its infancy and not much research has been done^94–96^. And, our groups discussed the crystal growth, crystal structure, thermal stabilities, structural geometries, and molecular dynamics of [NH_3_(CH_2_)_*n*_NH_3_]*M*Cl_4_ single crystals^97–113^. Despite the numerous applications of these compounds, there has been limited discussion about their structural dynamics and thermal properties due to variations in transition metal ions and methylene chain length.

In recent times, the potential of perovskites has spurred heightened research into analyzing their structural and mechanical properties. It has been noted that the commonly used CH_3_NH_3_PbI_3_ undergoes a phase transition at 329 K, falling within the operational temperature range of solar cells, and exhibits poor photostability. Consequently, ensuring the stability of perovskite solar cell devices emerges as a paramount concern. Moreover, despite their intriguing attributes, materials like perovskites decompose in humid air and pose toxicity risks due to lead. Hence, the imperative lies in developing hybrid perovskites that can be substituted with environmentally friendly alternatives.

This review delves into the potential applications of lead-free organic–inorganic perovskite-type crystals, specifically [NH_3_(CH_2_)_*n*_NH_3_]*M*Cl_4_ (*n* = 2, 3, 4, 5, and 6; *M* = Mn, Co, Cu, Zn, and Cd). We seek organic–inorganic hybrid perovskite materials characterized by high or no phase transition temperature, and thermal stability. Through examining the characteristics related to methylene length and the variation of transition metals, our aim is to identify enhanced materials meeting the aforementioned criteria. In this study, single crystals of organic–inorganic hybrid [NH_3_(CH_2_)_*n*_NH_3_]*M*Cl_4_ were grown via the aqueous solution method. We discuss their crystal structure, phase transition temperature (T_C_), and thermal decomposition temperature (T_d_). The ^1^H and ^13^C magic angle spinning (MAS) nuclear magnetic resonance (NMR) method plays a crucial role in understanding local dynamics. Determining the spin–lattice relaxation times T_1ρ_ for ^1^H and ^13^C is essential for studying dynamical processes. By analyzing the relaxation times of nuclei in different cationic environments, we gain detailed insights into their motion, particularly in the low- to mid-kHz frequency range. Furthermore, we consider the NMR spin–lattice relaxation times T_1ρ_, which reflect the energy transfer surrounding ^1^H and ^13^C atoms. Our results provide a comparative overview of the thermal stability of [NH_3_(CH_2_)_*n*_NH_3_]*M*Cl_4_, varying methylene length and metal ion as parameters. This review aims to advance the development of lead-free mixtures and the creation of a next-generation predictive model that combines both thermal stability and dynamic metal interactions.

## Methods

### Crystal growth

[NH_3_(CH_2_)_*n*_NH_3_]*M*Cl_4_ (*n* = 2, 3, 4, 5, and 6; *M* = Mn, Co, Cu, Zn, and Cd) single crystals were grown using the aqueous solution method. NH_2_(CH_2_)_*n*_NH_2_∙2HCl (Sigma-Aldrich) and *M*Cl_2_ (Sigma-Aldrich) were dissolved in distilled water. The mixture was stirred and heated, and the resulting solution was filtered. After a few weeks in a constant-temperature bath at 300 K, the single crystals were obtained.

### Characterization

At 300 K, the lattice constants were determined via a single-crystal X-ray diffraction (SCXRD) experiment conducted at the Korea Basic Science Institute (KBSI) Seoul Western Center. The experiment utilized a Bruker diffractometer equipped with a graphite-monochromated Mo-Kα target (D8 Venture PHOTON III M14) and a nitrogen cold flow (− 50 °C). Data collection was performed using SMART APEX3 (Bruker 2016) and analyzed with SAINT software (Bruker, 2016). The structure was refined using full-matrix least-squares on F^2^ with SHELXTL^114^. Hydrogen atoms were positioned according to the geometric arrangement within the single crystal structure. Additionally, powder X-ray diffraction (PXRD) patterns were obtained at various temperatures using an XRD system with a Mo-Kα radiation source, the same as that used in SCXRD.

Differential scanning calorimetry (DSC) experiments were conducted over the temperature range of 193 to 573 K, employing a heating rate of 10 °C/min under N_2_ gas. Thermogravimetric analysis (TGA) and differential thermal analysis (DTA) experiments, utilizing a thermogravimetric analyzer (TA Instrument), were performed within the temperature interval of 300 to 873 K, employing a heating rate of 10 °C/min under N_2_ gas as well.

The NMR spectra of the twenty-one crystals were acquired using a solid-state NMR spectrometer (AVANCE III, Bruker) operating at 400 MHz, located at the Seoul Western Seoul Center of the KBSI. The samples, placed in cylindrical zirconia rotors, were spun at a rate of 10 kHz for MAS NMR experiments. To determine the chemical shift, adamantane and tetramethylsilane (TMS) were used as standard materials for ^1^H and ^13^C, respectively, ensuring accurate NMR chemical shift measurements. MAS spin–lattice relaxation time T_1ρ_ values were measured using a π/2 − τ pulse with a spin-lock pulse of duration τ, and the π/2 pulse width was determined using a previously reported method^97–113^.

## Experimental results

### Crystal growth

**Figure 2:**
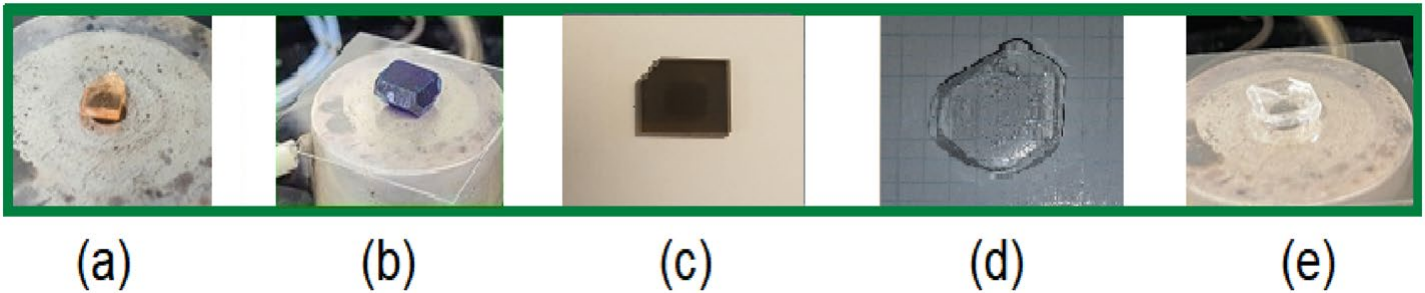
Morphology of (**a**) [NH_3_(CH_2_)_3_NH_3_]MnCl_4_, (**b**) [NH_3_(CH_2_)_3_NH_3_]CoCl_4_, (**c**) [NH_3_(CH_2_)_3_NH_3_]CuCl_4_, (**d**) [NH_3_(CH_2_)_3_NH_3_]ZnCl_4_, and (**e**) [NH_3_(CH_2_)_2_NH_3_]CdCl_4_ single crystals.

To obtain single crystals of [NH_3_(CH_2_)_*n*_NH_3_]*M*Cl_4_ (*n* = 2, 3, 4, 5, and 6; *M* = Mn, Co, Cu, Zn, and Cd), NH_2_(CH_2_)_n_NH_2_∙2HCl (Sigma-Aldrich) and *M*Cl_2_ (Sigma-Aldrich) were mixed in distilled water according to the molar ratio. The mixture was completely dissolved by stirring and heating to create a saturated solution. These prepared saturated solutions were then placed in a constant-temperature bath at 300 K and slowly evaporated to facilitate the growth of single crystals. Among them, crystals of (a) [NH_3_(CH_2_)_3_NH_3_]MnCl_4_, (b) [NH_3_(CH_2_)_3_NH_3_]CoCl_4_, (c) [NH_3_(CH_2_)_3_NH_3_]CuCl_4_, (d) [NH_3_(CH_2_)_3_NH_3_]ZnCl_4_ with *n* = 3, and (e) [NH_3_(CH_2_)_2_NH_3_]CdCl_4_ with *n* = 2 are depicted in Fig. 2. Mn crystals appear light brown, Co crystals are dark blue, Cu crystals are dark brown, and Zn and Cd crystals are colorless and transparent.

**Figure 3:**
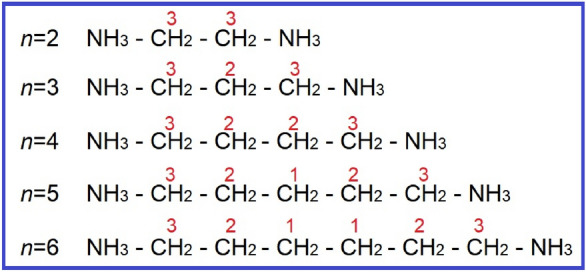
Notations of carbons according to the length in the cation structure of [NH_3_(CH_2_)_*n*_NH_3_]*M*Cl_4_ (*n* = 2, 3, 4, 5, and 6; *M* = Mn, Co, Cu, Zn, and Cd) crystals.

On the other hand, in the [NH_3_(CH_2_)_*n*_NH_3_]*M*Cl_4_ crystals (*n* = 2, 3, 4, 5, and 6; *M* = Mn, Co, Cu, Zn, and Cd), the positions of carbons along the lengths of *n* in the [NH_3_(CH_2_)_*n*_NH_3_] cation are indicated, as shown in Fig. 3. For *n* = 2, 3, and 4, the CH_2_ close to NH_3_ at both ends of the organic chain is denoted as CH_2_-3, the CH_2_ in the middle of two CH_2_-3 is designated as CH_2_-2. For *n* = 5 and 6, the CH_2_ close to NH_3_ is represented as CH_2_-3, the CH_2_ at the center is designated as CH_2_-1, and the CH_2_ in the middle of two CH_2_-3 and CH_2_-1 is denoted as CH_2_-2.

### [NH_3_(CH_2_)_n_NH_3_]MnCl_4_ (n = 2, 3, 4, and 5)

#### Crystal structures

**Figure 4:**
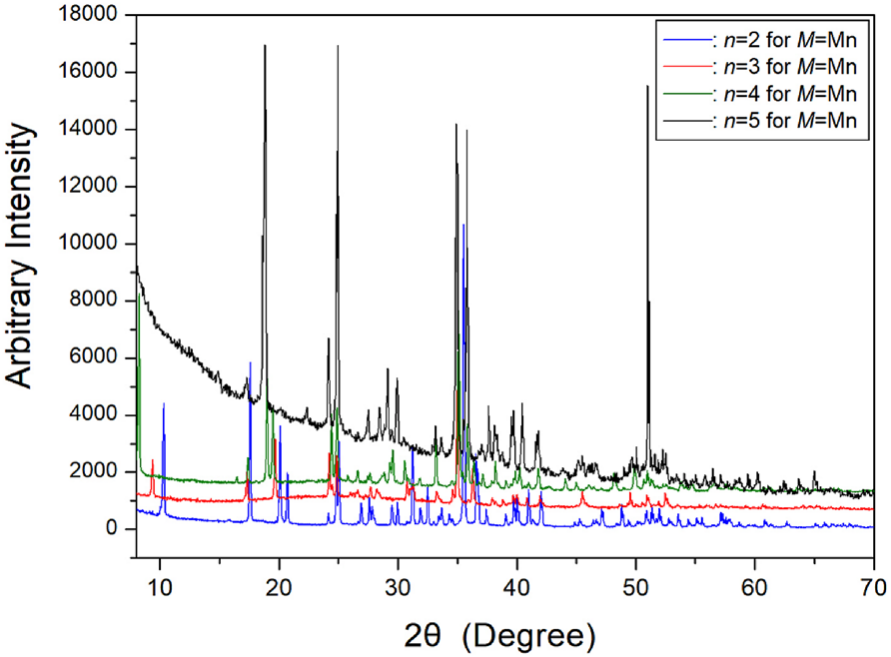
The powder X-ray diffraction patterns of [NH_3_(CH_2_)_*n*_NH_3_]MnCl_4_ (*n* = 2, 3, 4, and 5) at 300 K^97^.

The SCXRD and PXRD experiments for the [NH_3_(CH_3_)_*n*_NH_3_]MnCl_4_ crystals (*n* = 2, 3, 4, and 5) were conducted at 300 K. The PXRD patterns for the four crystals are depicted in Fig. 4. Based on the SCXRD results, the space group and lattice parameters for [NH_3_(CH_3_)_2_NH_3_]MnCl_4_ (*n* = 2) were determined as *P2*_*1*_*/c* with *a* = 8.614 (5) Å, *b* = 7.127 (3) Å, *c* = 7.188 (3) Å, *β* = 92.772 (28)°, and Z = 2^64,97^. Similarly, for [NH_3_(CH_3_)_3_NH_3_] MnCl_4_ (*n* = 3), the space group was identified as *Imma*, and the lattice parameters were determined as *a* = 19.009 (3) Å, *b* = 7.169 (1) Å, *c* = 7.357 (1) Å, with Z = 4^9,67^. In the case of [NH_3_(CH_3_)_4_NH_3_]MnCl_4_ (*n* = 4), the space group and lattice parameters were found to be *P2*_*1*_*/c*, with *a* = 10.826 (4) Å, *b* = 7.178 (3) Å, *c* = 7.312 (3) Å, *β* = 92.605 (14)°, and Z = 2^63,97^. Additionally, the crystal structure of [NH_3_(CH_3_)_5_NH_3_]MnCl_4_ adopts an orthorhombic structure, with lattice constants *a* = 24.004 Å, *b* = 7.190 Å, *c* = 7.395 Å, and Z = 4 in the space group *Imma*^9,98^. As shown in Fig. 1a, the Mn atom is coordinated to six Cl atoms, forming an almost regular octahedron, MnCl_6_. Furthermore, the six N-linked hydrogen atoms in one formula unit form N − H∙∙∙Cl hydrogen bonds. The structures, space groups, and lattice constants of [NH_3_(CH_3_)_*n*_NH_3_]MnCl_4_ (*n* = 2, 3, 4, and 5) crystals are summarized in Table 1. It is noteworthy that the crystal structures are monoclinic when *n* is even and orthorhombic when *n* is odd.

**Table 1:** The structures, space groups, and lattice constants (Å) of [NH_3_(CH_2_)_*n*_NH_3_]MnCl_4_ (*n* = 2, 3, 4, and 5) crystals.

*n*	2	3	4	5
Structure	Monoclinic	Orthorhombic	Monoclinic	Orthorhombic
Space group	*P2*_*1*_*/c*	*Imma*	*P2*_*1*_*/*$c$	*Imma*
Lattice constants				
a (Å)	8.614	19.009	10.826	24.004
*b* (Å)	7.127	7.169	7.178	7.190
*c* (Å)	7.188	7.357	7.312	7.395
*β* (^∘^)	92.772		92.605	
*Z*	2	4	2	4
References	^64,97^	^67,97^	^63,97^	^9,98^

#### Phase transition temperatures

**Figure 5:**
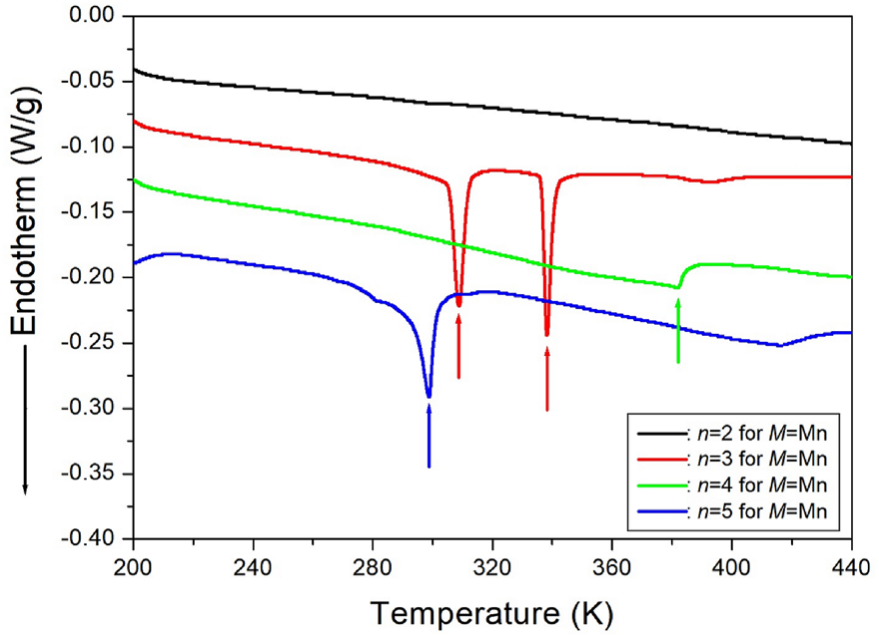
The differential scanning calorimetry curves of [NH_3_(CH_2_)_*n*_NH_3_]MnCl_4_ (*n* = 2, 3, 4, and 5) crystals^97,98^.

The DSC curves in Fig. 5 depict the thermal behavior of four crystals under a heating rate of 10 °C/min. When *n* = 2, no peaks were observed^97^. However, for *n* = 3, two distinct endothermic peaks indicative of phase transition temperatures were observed at 308 K and 338 K. In the case of *n* = 4, a minor endothermic peak was observed at 378 K. Conversely, for *n* = 5, an endothermic peak was observed at 298 K^98^. The identified endothermic peaks from the DSC curves were further confirmed as phase transition temperatures through PXRD patterns and polarizing microscopy experiments in response to temperature changes^97,98^.

#### Thermodynamic properties

**Figure 6:**
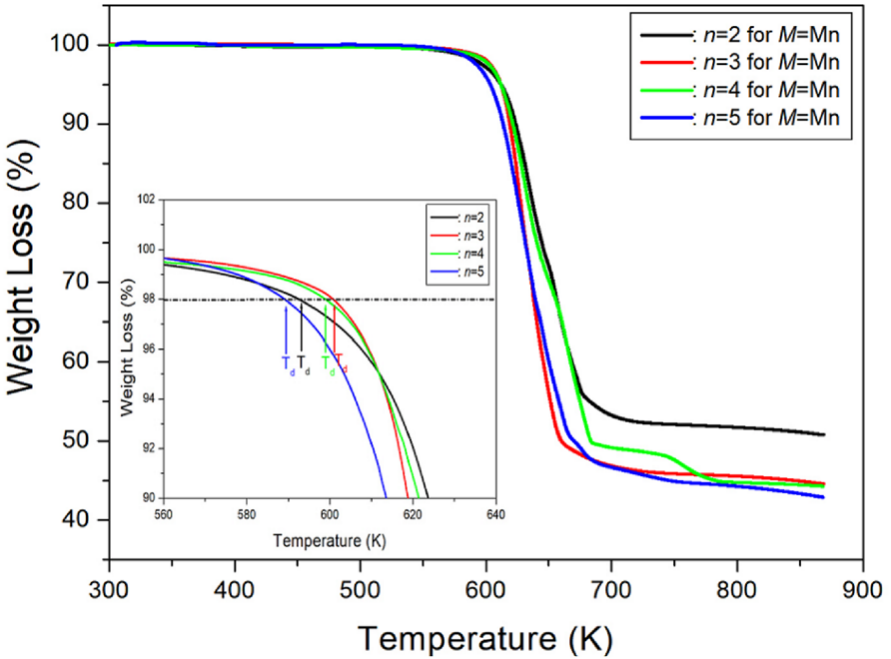
The thermogravimetry analysis curves of [NH_3_(CH_2_)_*n*_NH_3_]MnCl_4_ (*n* = 2, 3, 4, and 5)^97,98^.

To investigate the thermal stabilities, TGA experiments were conducted with a heating rate of 10 °C/min, mirroring the conditions of the DSC experiment. The TGA curves presented in Fig. 6 reveal that crystals with *n* = 2, 3, 4, and 5 exhibit thermal stability up to approximately 593, 600, 598, and 589 K, respectively. This stability is defined as the thermal decomposition temperature (T_d_) corresponding to a 2% weight loss^97,98^. It is noteworthy that these crystals initiate weight loss at higher temperatures. For *n* = 2, weight losses of 14 and 31% around 628 and 654 K are attributed to the partial thermal decomposition of HCl and 2HCl moieties, respectively. The temperatures at which partial decomposition occurs for HCl and 2HCl in *n* = 3 and *n* = 4 are comparable to those observed for *n* = 2. In the case of *n* = 5, mass losses of approximately 12 and 24% around 617 and 630 K may be attributed to the loss of HCl and 2HCl, respectively. For methylene lengths of 3, 4, and 5, weight losses of 55% are observed above 700 K, whereas a weight loss of about 48% is seen for *n* = 2. In summary, these four crystals exhibit relatively high stability at elevated temperatures.

#### MAS NMR chemical shifts and spin–lattice relaxation times

**Figure 7:**
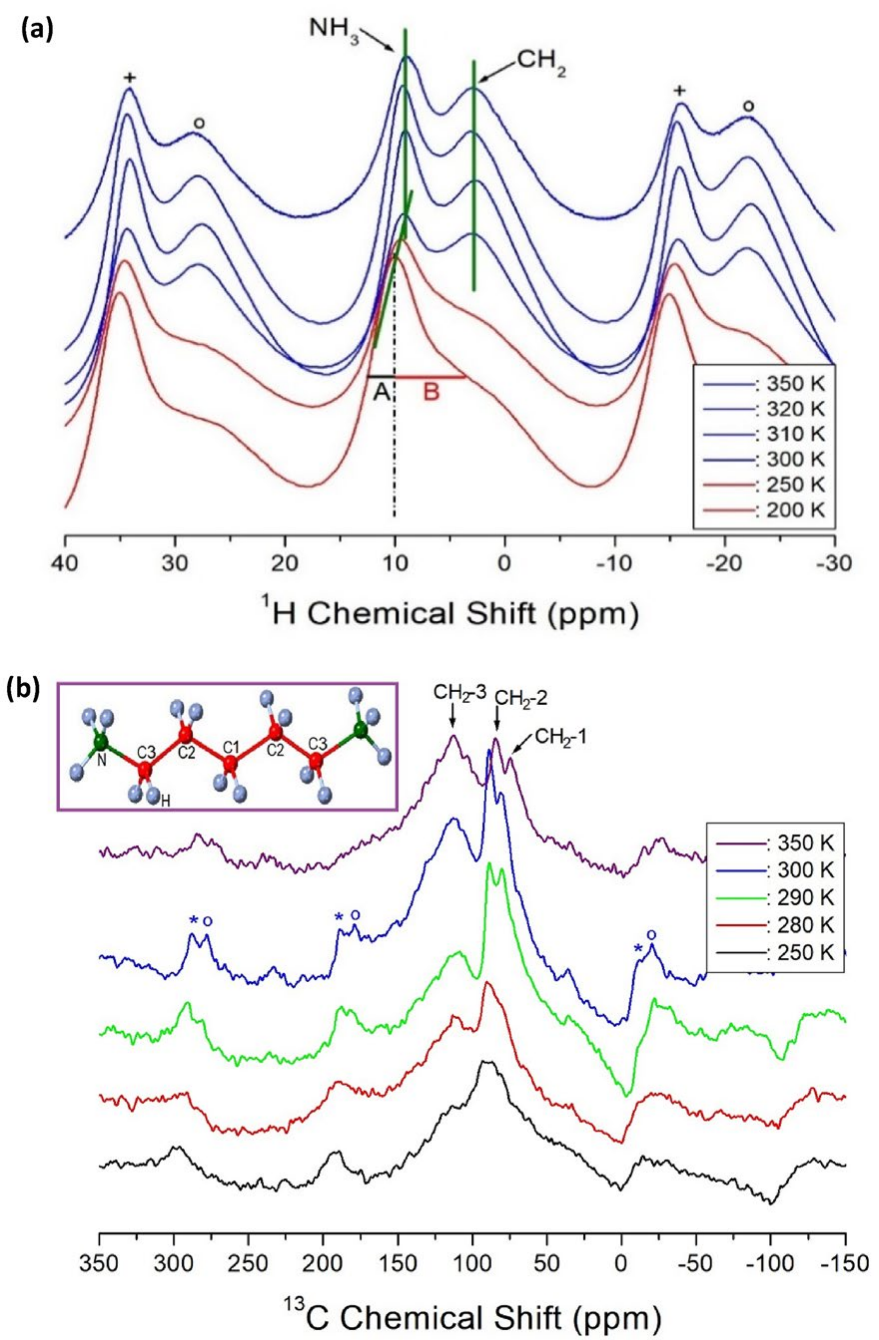
(**a**) ^1^H NMR chemical shifts of [NH_3_(CH_2_)_5_NH_3_]MnCl_4_ at 200, 250, 300, 310, 320, and 350 K. + and o are the spinning sidebands for NH_3_ and CH_2_, respectively^98^. (**b**) ^13^C NMR chemical shifts of [NH_3_(CH_2_)_5_NH_3_]MnCl_4_ at 250, 280, 290, 300, and 350 K. o and * are the spinning sidebands for CH_2_-1 and CH_2_-2, respectively^98^.

The ^1^H NMR spectra of [NH_3_(CH_2_)_*n*_NH_3_]MnCl_4_ (*n* = 2, 3, 4, and 5) crystals were measured using NMR spectroscopy at various temperatures. Figure 7a displays the ^1^H NMR chemical shifts for [NH_3_(CH_2_)_5_NH_3_]MnCl_4_ with *n* = 5. The resonance lines observed at lower temperatures are asymmetric due to the overlap of signals representing NH_3_ and CH_2_. The line widths denoted by A and B on the left and right sides of the half-maximum in Fig. 7a are not equal. Above 300 K, the NH_3_ and CH_2_ signals are resolved, with chemical shifts of 9.29 and 2.89 ppm, respectively. Spinning sidebands are marked with + and o to represent ^1^H in NH_3_ and CH_2_, respectively. The ^1^H chemical shifts of CH_2_ show minimal variation near T_C_ (= 298 K), while changes in the ^1^H chemical shifts of NH_3_ are observed at approximately T_C_. The more significant changes in the ^1^H NMR chemical shifts of NH_3_ compared to those in the ^1^H NMR chemical shifts of CH_2_ near T_C_ suggest a modification in the N − H⋯Cl hydrogen bonding between Cl around Mn and H of NH_3_.

In addition, the ^13^C NMR chemical shifts in the MAS NMR spectra of CH_2_ in [NH_3_(CH_2_)_5_NH_3_]MnCl_4_ were recorded near T_C_. The ^13^C signal of TMS was observed at 38.3 ppm at 300 K; thus, 38.3 ppm was set as the standard for the ^13^C chemical shifts. Here, CH_2_-1 in the [NH_3_(CH_2_)_5_NH_3_] cation is located at the center of the cation, CH_2_-3 is positioned adjacent to the NH_3_ in the cation, and CH_2_-2 is situated between CH_2_-1 and CH_2_-3, as shown in the inset of Fig. 7b. The respective chemical shifts of CH_2_-1, CH_2_-2, and CH_2_-3 at 300 K are 80.96, 88.92, and 113.44 ppm, as illustrated in Fig. 7b. The o and * symbols represent the spinning sidebands for CH_2_-1 and CH_2_-2. The ^13^C chemical shifts of CH_2_-3 do not vary significantly near T_C_, whereas those of CH_2_-1 and CH_2_-2 show variations near T_C_.

The ^1^H and ^13^C MAS NMR spectra were acquired at various delay times for each temperature. The signal intensities in the NMR spectrum, corresponding to different delay times, are modeled as an exponential function. The magnetization decay rates for protons and carbon are characterized by T_1ρ_ as shown below^115^:

1 $${\text{P}}\left( \tau \right)/{\text{P}}\left( 0 \right) \, = {\text{ exp}}\left( { - \tau /{\text{T}}_{{{1}\rho }} } \right),$$

**Figure 8:**
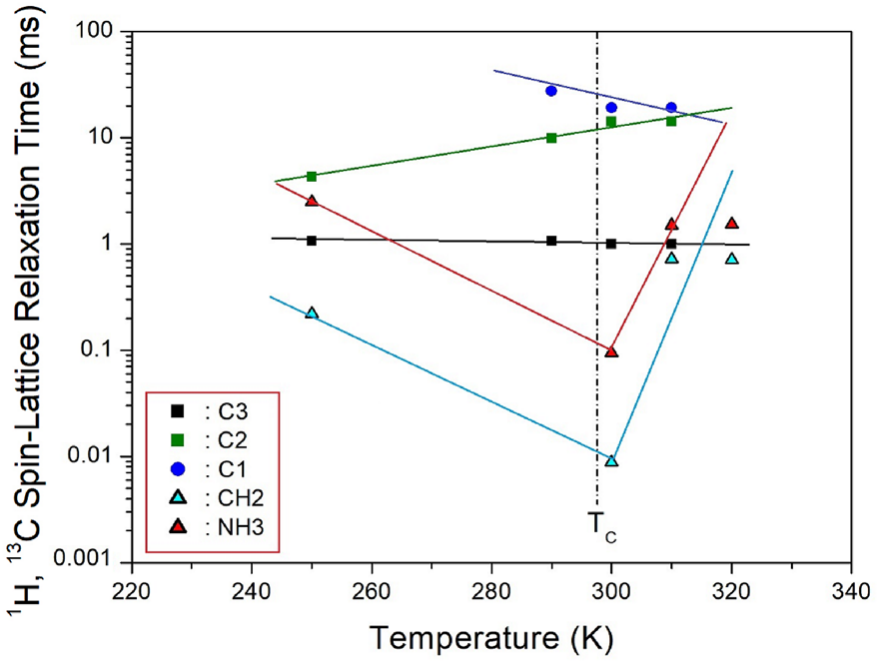
The ^1^H and ^13^C spin–lattice relaxation times in [NH_3_(CH_2_)_5_NH_3_]MnCl_4_ with increasing temperatures^98^.

Here, P(*τ*) and P(0) represent the NMR signal intensities at delay time *τ* and *τ* = 0, respectively. The ^1^H and ^13^C NMR spectra were recorded with various time delays, and the decay curves can be represented by a single exponential function as described in Eq. (1). The ^1^H T_1ρ_ values for NH_3_ and CH_2_ at 300 K were notably short, measuring 20.8, 15.4, and 14.4 ms for 2, 3, and 4, respectively^97^. For a methylene length of *n* = 5, the ^1^H T_1ρ_ values at 300 K were 94 μs for NH_3_ and 8.86 μs for CH_2_, respectively. These ^1^H T_1ρ_ values exhibit a strong dependence on temperature, as illustrated in Fig. 8. The ^1^H T_1ρ_ values of CH_2_ and NH_2_ undergo significant changes near T_C_, suggesting a substantial alteration in the ^1^H energy transfer of CH_2_ and NH_3_.

Additionally, the ^13^C T_1ρ_ values of CH_2_-1, CH_2_-2, and CH_2_-3 in the case of *n* = 5 are determined from the slopes of their recovery traces. The ^13^C T_1ρ_ values near T_C_ exhibit virtual continuity^98^. Notably, the T_1ρ_ values of CH_2_-3, located adjacent to NH_3_, are the shortest. The relatively small T_1ρ_ values of CH_2_-3, in proximity to Mn^2+^ ions, are associated with the magnetic moments of the paramagnetic Mn^2+^ ions.

### [NH_3_(CH_2_)_*n*_NH_3_]CoCl_4_ (*n* = 3 and 5)

#### Crystal structures

**Figure 9:**
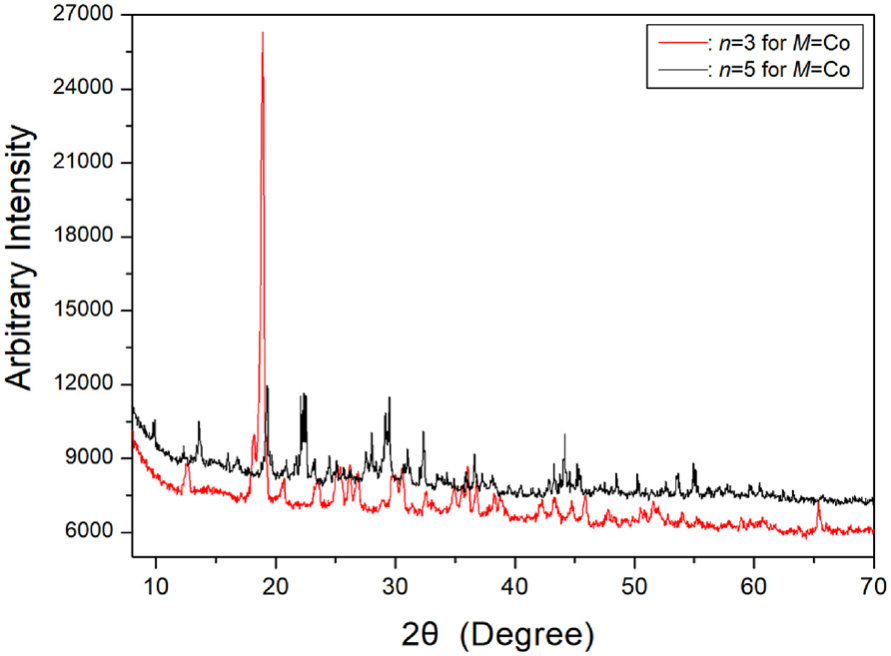
The powder X-ray diffraction patterns of [NH_3_(CH_2_)_*n*_NH_3_]CoCl_4_ (*n* = 3 and 5) at 300 K^99^.

The structures of [NH_3_(CH_2_)_*n*_NH_3_]CoCl_4_ crystals (*n* = 3 and 5) were determined through SCXRD and PXRD experiments conducted at room temperature. Additionally, the PXRD patterns for *n* = 3 and 5 at 300 K are presented in Fig. 9. And, the space group and lattice constants of the [NH_3_(CH_2_)_3_NH_3_]CoCl_4_ crystal with *n* = 3 were determined as *P2*_*1*_*/c* with *a* = 10.738 (40) Å, *b* = 10.712 (36) Å, *c* = 10.926 (39) Å, *β* = 119.216 (266)°, and Z = 4^72,99^. Similarly, the space group and lattice constants of [NH_3_(CH_2_)_5_NH_3_]CoCl_4_ with *n* = 5 were identified as *P2*_*1*_*/c*, with *a* = 7.167 (1) Å, *b* = 15.949 (2) Å, *c* = 11.145 (1) Å, *β* = 98.497 (5)°, and Z = 4^73,96^. The structures and space groups of the two crystals resembled monoclinic (*P2*_*1*_*/c*), as detailed in Table 2. For odd values of *n*, the crystal structure of [NH_3_(CH_2_)_*n*_NH_3_]MnCl_4_ is orthorhombic, while the crystal structure of [NH_3_(CH_2_)_*n*_NH_3_]CoCl_4_ is monoclinic.

**Table 2:** The structures, space groups, and lattice constants (Å) of [NH_3_(CH_2_)_*n*_NH_3_]CoCl_4_ (*n* = 3 and 5) crystals.

*n*	3	5
Structure	Monoclinic	Monoclinic
Space group	*P2*_*1*_*/c*	*P2*_*1*_*/*$c$
Lattice constants		
a (Å)	10.738	7.167
*b* (Å)	10.712	15.949
*c* (Å)	10.926	11.145
*β* (^∘^)	119.216	98.497
*Z*	4	4
References	^72,99^	^73,99^

#### Phase transition temperatures

**Figure 10:**
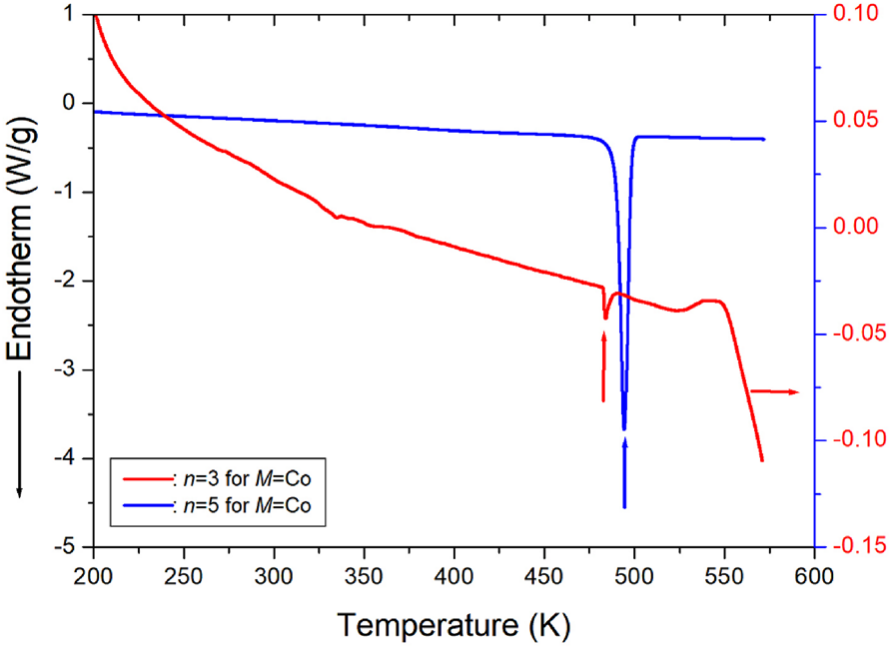
The differential scanning calorimetry curves of [NH_3_(CH_2_)_*n*_NH_3_]CoCl_4_ (*n* = 3 and 5) crystals^99^.

In the DSC thermogram of [NH_3_(CH_2_)_3_NH_3_]CoCl_4_ with *n*=3, only one weak endothermic peak was observed at 483 K, as shown in Fig. 10^99^. Meanwhile, in [NH_3_(CH_2_)_5_NH_3_]CoCl_4_ with *n*=5, a peak was observed at 494 K. To accurately confirm whether the observed peaks in the two crystals were melting or phase transition temperatures, PXRD results were obtained according to temperature changes.^99^ These peaks were confirmed to be phase transition temperatures.

#### Thermodynamic properties

**Figure 11:**
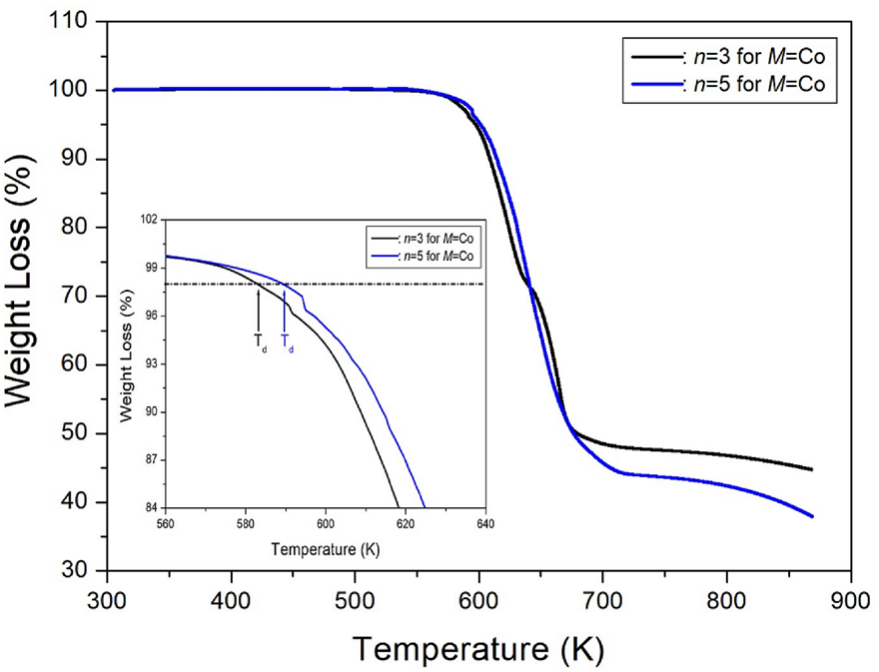
The thermogravimetry analysis curves of [NH_3_(CH_2_)_*n*_NH_3_]CoCl_4_ (*n* = 3 and 5)^99^.

To understand the thermodynamic properties of these crystals, TGA was performed at a heating rate of 10 °C/min, similar to the procedure used in the DSC experiments. The TGA curves for crystals with *n*=3 and 5 are presented in Fig. 11. The molecular weights of both crystals decreased as the temperature increased. The amount of residue at higher temperatures was calculated based on the total molecular weight. By considering the number of CH_2_ units in the methylene length, the molecular weight loss at approximately 583 and 589 K for *n*=3 and 5, respectively, marked the onset of partial thermal decomposition, with 2 % weight loss set as T_d_. For *n*=3, weight losses of approximately 13 and 26 % around 614 and 633 K could be attributed to thermal decomposition and the partial escape of HCl and 2HCl moieties. The temperatures corresponding to the partial escape of HCl and 2HCl moieties for *n*=5 were almost similar to those for *n* = 3, with weight losses of approximately 12 and 24 % near 614 and 632 K due to thermal decomposition and the partial escape of HCl and 2HCl moieties, respectively^99^. The molecular weights of both crystals sharply decreased between 600 and 700 K, with a 50~55% weight loss occurring at approximately 700 K.

#### MAS NMR chemical shifts and spin-lattice relaxation times

**Figure 12:**
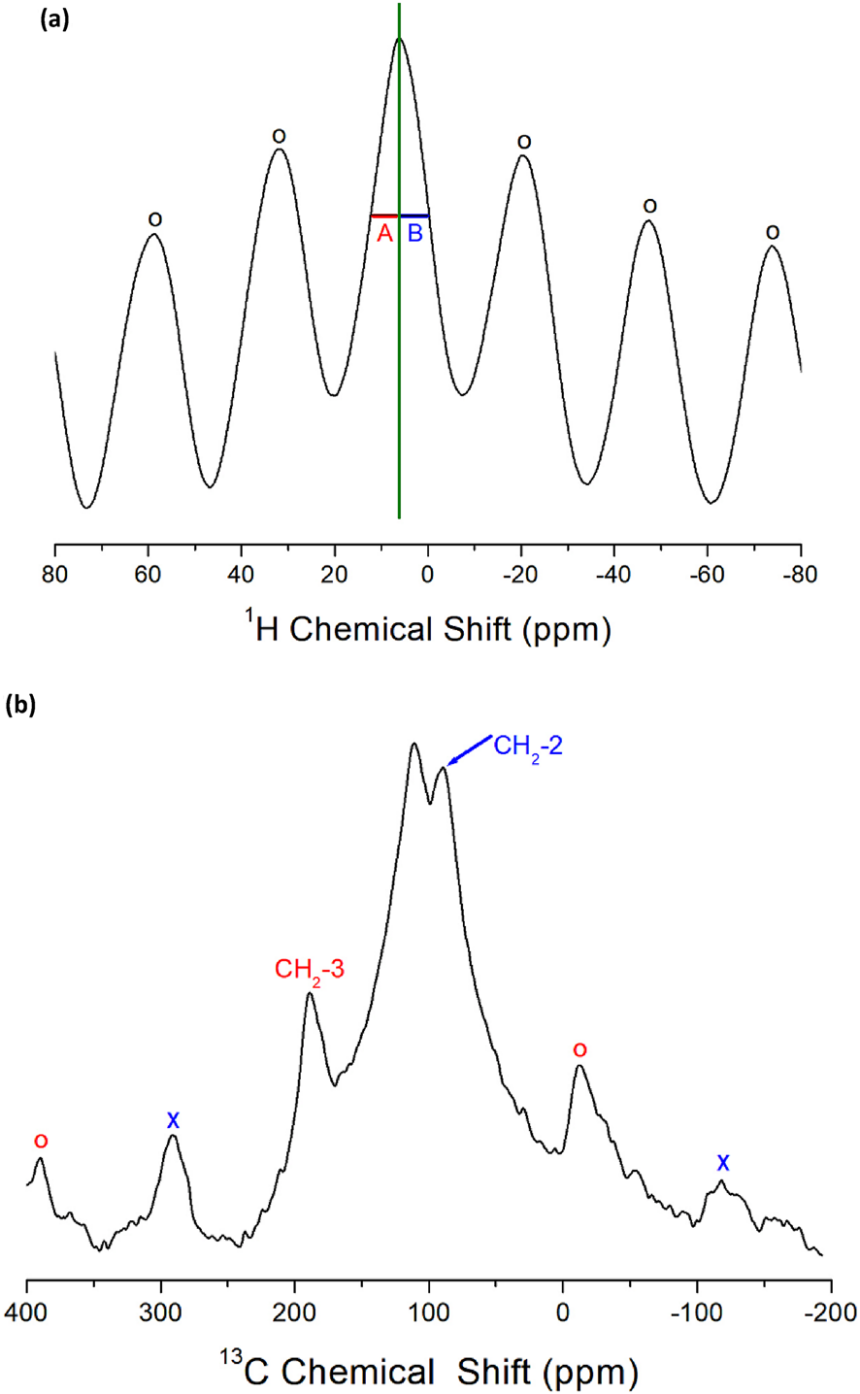
(**a**) The ^1^H NMR chemical shifts of [NH_3_(CH_2_)_3_NH_3_]CoCl_4_ at 300 K. The open circles are sidebands^99^. (**b**) The ^13^C NMR chemical shifts of [NH_3_(CH_2_)_3_NH_3_]CoCl_4_ at 340 K. The crosses and open circles are sidebands for CH_2_-2 and CH_2_-3, respectively^99^.

The ^1^H NMR spectra of [NH_3_(CH_2_)_3_NH_3_]CoCl_4_ crystals recorded by MAS NMR experiment at 300 K were represented in Fig. 12a. The chemical shift of the resonance peak was observed at 6.40 ppm as a single resonance line. The spinning sideband is marked with open circles. The observed resonance line was symmetric, corresponding to the overlapping lines of NH_3_ and CH_2_; the line widths denoted by A and B on the left and right sides of the half-maximum are identical. Thus, the chemical shifts of NH_3_ and CH_2_ did not separate and completely overlapped.

Moreover, the MAS ^13^C NMR chemical shifts of CH_2_ in [NH_3_(CH_2_)_3_NH_3_]CoCl_4_ were recorded at 300 K. The NMR signal at 115.38 ppm shows the ^13^C baseline signal in the absence of a sample, and it was not considered. The chemical shifts at 340 K were observed at 91.65 and 188.25 ppm for CH_2_-2 and CH_2_-3, as shown in Fig. 12b. As shown in Fig. 3, CH_2_-1 is located in the center of the [NH_3_(CH_2_)_3_NH_3_] cation, while CH_2_-3 is located near NH_3_ in the cation. The crosses and open circles indicate the spinning sidebands for CH_2_-2 and CH_2_-3, respectively.

**Figure 13:**
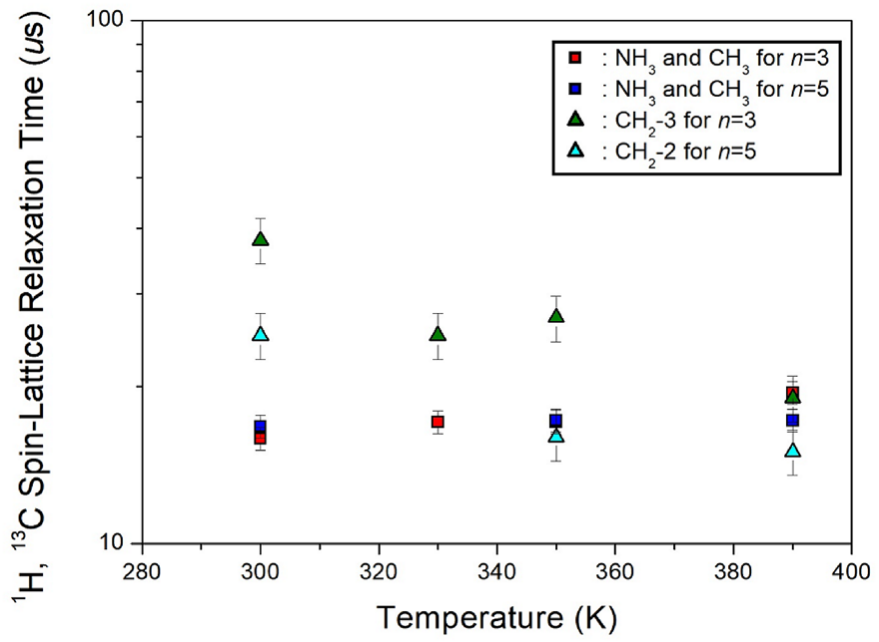
The ^1^H and ^13^C spin–lattice relaxation times in [NH_3_(CH_2_)_*n*_NH_3_]CoCl_4_ (*n* = 3 and 5) with increasing temperatures^99^.

The MAS ^1^H NMR spectra of NH_3_(CH_2_)_*n*_NH_3_CoCl_4_ (*n*=3 and 5) were recorded, capturing signal intensities at various delay times ranging from 1 *μs* to 5 *ms*. The ^1^H T_1ρ_ values, determined from the slopes of the decay rate of proton magnetization, were found to be short, specifically 15.89–19.45 *μs* for *n*=3 and 16.76–17.22 *μs* for *n*=5, with respect to changes in temperature. Notably, these values for *n*=3 and 5 were nearly identical and temperature-independent, as illustrated in Fig. 13. Here, the ^1^H T_1ρ_ values for NH_3_ and CH_2_ could not be distinguished due to the overlapping ^1^H signals of NH_3_ and CH_2_.

In contrast, the intensities of the ^13^C NMR spectra were measured at various delay times, ranging from 1 *μs* to 10 *ms*. The ^13^C T_1ρ_ values for CH_2_-2 and CH_2_-3 were obtained from the slope of their recovery traces. Due to the ^13^C baseline being closer to CH_2_-2 for *n*=3 and CH_2_-3 for *n*=5, obtaining accurate T_1ρ_ values was challenging. The ^13^C T_1ρ_ values were determined to be 38–19 *μs* for CH_2_-3 in the case of *n*=3 and 25–15 *μs* for CH_2_-2 in the case of *n*=5, considering the change in temperature. In both crystals, the ^13^C T_1ρ_ values exhibited a slightly shorter value as the temperature increased, suggesting the activation of molecular motion. Notably, the ^1^H and ^13^C T_1ρ_ values of the two crystals with paramagnetic Co^2+^ ions were much shorter than the values of crystals without paramagnetic ions. These results align well with the observation that T_1ρ_ is closely related to paramagnetic ions, being inversely proportional to the square of the magnetic moment^99^.

### [NH_3_(CH_2_)_*n*_NH_3_]CuCl_4_ (*n*=2, 3, 4, 5 and 6)

#### Crystal structures

**Figure 14:**
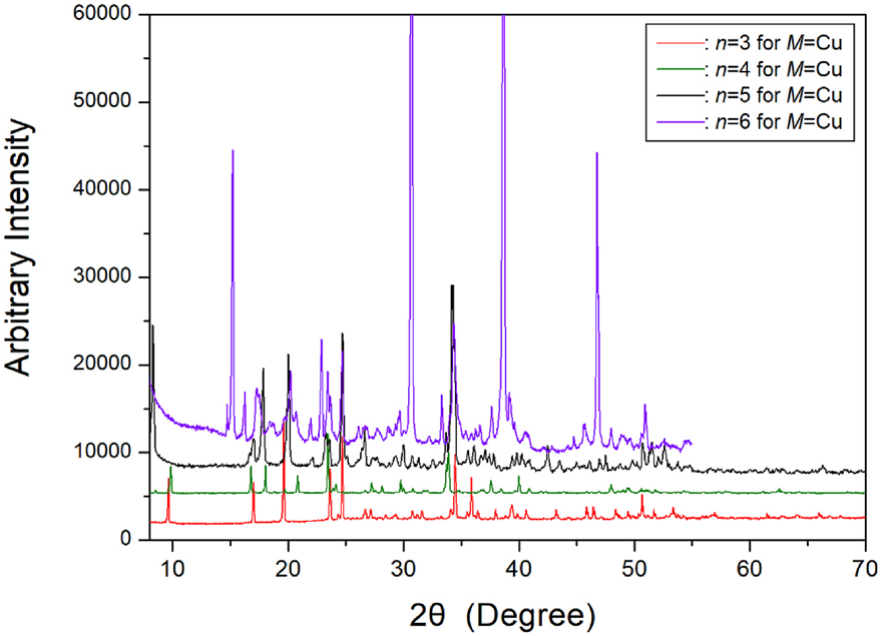
The powder X-ray diffraction patterns of [NH_3_(CH_2_)_*n*_NH_3_]CuCl_4_ (*n* = 3, 4, 5, and 6) at 300 K^101,103,104^.

The PXRD patterns for [NH_3_(CH_2_)_*n*_NH_3_]CuCl_4_ (*n*=2, 3, 4, 5 and 6) were obtained at 300 K, as shown in Fig. 14. According to the previously reported case for *n*=2, the crystal at room temperature exhibits a monoclinic structure with the space group *P2*_*1*_*/b* and Z=2. The unit cell parameters are *a*=8.109 Å, *b*=7.158 Å, *c*=7.363 Å, and *γ*=92.37°^64,74^. Based on our XRD experimental results, the [NH_3_(CH_2_)_3_NH_3_]CuCl_4_ crystal with *n*=3 has an orthorhombic structure with the space group *Pnma*. The cell parameters are *a*=7.202 Å, *b*=18.260 Å, *c*=7.515 Å, and Z=4^78,101^. Additionally, the crystal structure of [NH_3_(CH_2_)_4_NH_3_]CuCl_4_ with *n*=4 is monoclinic, corresponding to space group *P2*_*1*_*/c*. The unit cell parameters are *a*=9.270 Å, *b*=7.600 Å, *c*=7.592 Å, *β*=103.14˚, and Z=2^75,76,102^. Furthermore, the crystal structure with *n*=5 is monoclinic, and the lattice constants analyzed from the SCXRD result were *a*=7.7385 Å, *b*=7.2010 Å, *c*=21.5308 Å, *β*=98.493°, and Z=4, with the space group *P2*_*1*_*/c*^79,103^. Finally, the SCXRD result for the [NH_3_(CH_2_)_6_NH_3_]CuCl_4_ crystal was obtained at 300 K; the hybrid was crystallized as a monoclinic structure with the space group *P2*_*1*_*/n* and lattice constants *a*=7.2224 Å, *b*=7.6112 Å, *c*=23.3315 Å, *β*=91.930°, and Z=4^104^. These structures consist of parallel 2D sheets of perovskite-type layers of corner-sharing CuCl_6_ octahedra interleaved by layers of [NH_3_(CH_2_)_6_NH_3_] cations that are nearly perpendicular to the layers. The structures, space groups, and lattice constants of [NH_3_(CH_2_)_*n*_NH_3_]CuCl_4_ (*n*=2, 3, 4, 5, and 6) crystals are summarized in Table 3.

**Table 3:** The structures, space groups, and lattice constants (Å) of [NH_3_(CH_2_)_*n*_NH_3_]CuCl_4_ (*n* = 2, 3, 4, 5, and 6) crystals.

*n*	2	3	4	5	6
Structure	Monoclinic	Orthorhombic	Monoclinic	Monoclinic	Monoclinic
Space group	*P2*_*1*_*/b*	*Pnma*	*P2*_*1*_*/c*	*P2*_*1*_*/c*	*P2*_*1*_*/n*
Lattice constants					
a (Å)	8.109	7.202	9.270	7.7385	7.2224
*b* (Å)	7.158	18.260	7.600	7.2010	7.6112
*c* (Å)	7.363	7.515	7.592	21.5308	23.3315
*β* (^∘^)	92.37		103.14	98.493	91.930
*Z*	2	4	2	4	4
References	^64,74^	^78,101^	^75,76,102^	^79,103^	^104^

#### Phase transition temperatures

**Figure 15:**
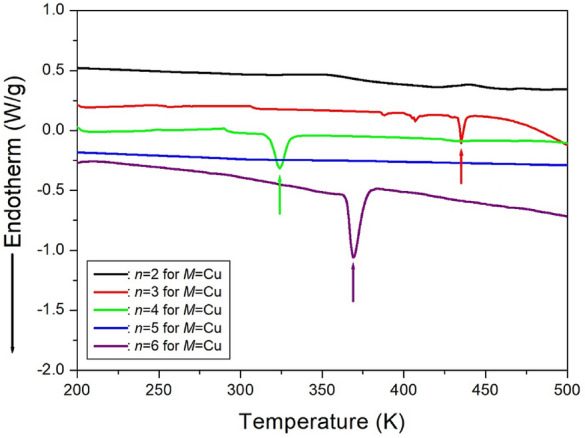
The differential scanning calorimetry curves of [NH_3_(CH_2_)_*n*_NH_3_]CuCl_4_ (*n* = 2, 3, 4, 5, and 6) crystals^100–104^.

The results of the DSC analysis of [NH_3_(CH_2_)_*n*_NH_3_]CuCl_4_ (*n*=2, 3, 4, 5, and 6) under a nitrogen atmosphere are presented in Fig. 15. The crystals [NH_3_(CH_2_)_2_NH_3_]CuCl_4_ and [NH_3_(CH_2_)_5_NH_3_]CuCl_4_ with *n*=2 and 5 do not undergo a phase transition^100,103^. However, an endothermic peak at 434 K was observed in the case of *n*=3, consistent with the structural phase transition^101^. Additionally, an endothermic peak corresponding to the phase transition of the [NH_3_(CH_2_)_4_NH_3_]CuCl_4_ crystal was detected at 323 K^101^. Finally, upon heating, an endothermic peak was observed for the DSC experiment on the [NH_3_(CH_2_)_6_NH_3_]CuCl_4_ crystal at 363 K^104^.

#### Thermodynamic properties

**Figure 16:**
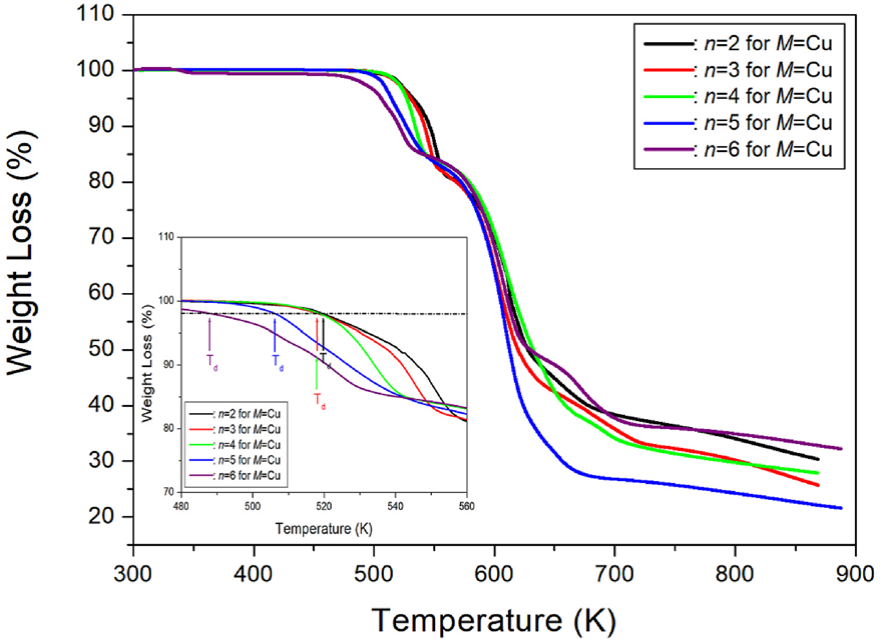
The thermogravimetry analysis curves of [NH_3_(CH_2_)_*n*_NH_3_]CuCl_4_ (*n* = 2, 3, 4, 5, and 6)^100–104^.

The measured TGA curves for [NH_3_(CH_2_)_n_NH_3_]CuCl_4_ (*n*=2, 3, 4, 5, and 6) are depicted in Fig. 16. The TGA curves reveal that the crystals with *n*=2, 3, 4, 5, and 6 exhibit thermal stability up to approximately 520, 518, 518, 506, and 488 K, respectively^100–104^. For *n*=2, weight losses of 14 and 27 % near 552 and 594 K, respectively, were attributed to the thermal decomposition of HCl and 2HCl. In the case of *n*=3, thermal stability was observed up to around 518 K; however, signs of weight loss above this temperature Indicated partial thermal decomposition. The compound [NH_3_(CH_2_)_3_NH_3_]CuCl_4_ with *n*=3 undergoes decomposition at high temperatures, with a 13 % weight loss near 539 K attributed to the decomposition of HCl moieties. The weight loss decreases rapidly between 500 and 600 K, with a 65 % weight loss occurring around 650 K. Similarly, for *n*=4, a stable state is observed up to 518 K, but partial thermal decomposition is observed at higher temperatures, resulting in a 12% weight loss around 538 K. In the case of [NH_3_(CH_2_)_5_NH_3_]CuCl_4_, the initiation of partial thermal decomposition, indicated by the first occurrence of molecular weight loss, occurs at approximately 506 K. As the temperature increases, the molecular weight decreases, with losses of 12 and 24% calculated from the total molecular weight attributed to the decomposition of HCl and 2HCl, respectively, and a weight loss of 80% at ~900 K. Finally, for *n*=6, mass loss begins at approximately 488 K, representing the T_d_. The molecular weight loss of [NH_3_(CH_2_)_6_NH_3_]CuCl_4_ occurs with increasing temperature, with losses of 11 and 23% calculated from the molecular weight attributed to the decomposition of HCl and 2HCl, respectively, and a weight loss of 70% occurs near 900 K.

#### MAS NMR chemical shifts and spin-lattice relaxation times

**Figure 17:**
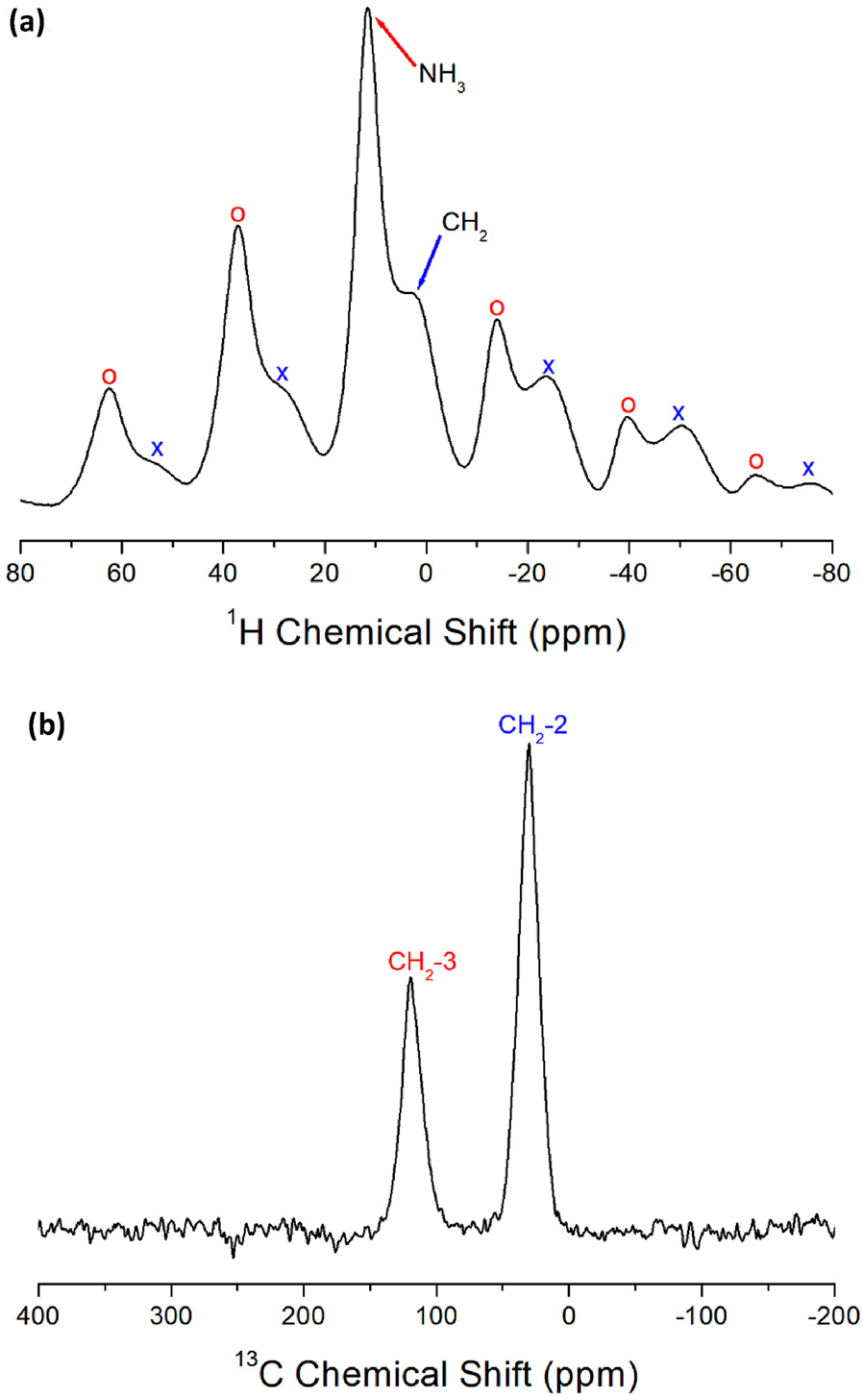
(**a**) The ^1^H NMR chemical shifts of [NH_3_(CH_2_)_3_NH_3_]CuCl_4_ at 300 K. The open circles and crosses are sidebands for NH_3_ and CH_2_, respectively^101^. (**b**) The ^13^C NMR chemical shifts of [NH_3_(CH_2_)_3_NH_3_]CuCl_4_ at 300 K^101^.

The chemical shift of the ^1^H NMR spectrum of [NH_3_(CH_2_)_3_NH_3_]CuCl_4_ crystals with *n*=3 was obtained, as illustrated in Fig. 17a. Two peaks in the NMR spectra are designated as NH_3_ and CH_2_, and the ^1^H signals from NH_3_ and CH_2_ were observed to slightly overlap. The spinning sidebands for CH_2_ are marked with crosses, and those for NH_3_ are denoted with open circles. At 300 K, the NMR chemical shift of ^1^H for CH_2_ was recorded at δ=2.76 ppm, whereas that for NH_3_ was observed at δ=11.48 ppm. According to the previous results from our group, the ^1^H peak for CH_2_ did not significantly change as the temperature increased, while for NH_3_, the chemical shift was temperature-dependent^101^.

The ^13^C NMR chemical shifts for CH_2_ in the crystals with *n*=2, 3, 4, 5, and 6 were measured as the temperature increased, and the ^13^C chemical shift at 300 K in the case of *n*=3 is shown in Fig. 17b. At 300 K, two resonance peaks were obtained at chemical shifts of δ=28.78 for CH_2_-2 and δ=124.97 ppm for CH_2_-3. The ^13^C chemical shifts for CH_2_-2 were different, being far away from NH_3_, while CH_2_-3 was close to NH_3_.

**Figure 18:**
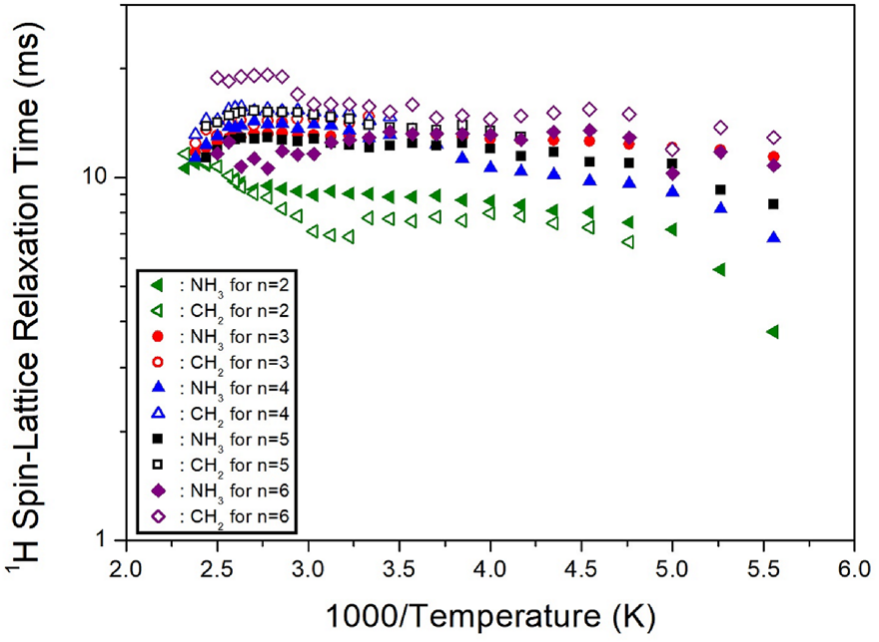
The ^1^H spin–lattice relaxation times in [NH_3_(CH_2_)_*n*_NH_3_]CuCl_4_ (*n* = 2, 3, 4, 5, and 6) as a function of inverse temperature^100–104^.

The ^1^H NMR spectra were also obtained with several delay times at each temperature, and the intensities of NMR spectra as a function of delay time were represented by a single exponential function. From the slope of the intensity vs. delay times curve, ^1^H T_1ρ_ values were obtained for the CH_2_ and NH_3_ peaks. For methylene lengths *n*=2, 3, 4, 5, and 6, ^1^H T_1ρ_ values at 300 K showed similar values in the range of 7–15 *ms*. The ^1^H T_1ρ_ values for *n*=2, 3, 4, 5, and 6 are shown in Fig. 18 as a function of inverse temperature. The ^1^H T_1ρ_ values were almost temperature-independent and were in the order of 10 *ms*. Here, the T_1ρ_ values were compared according to the cation length from *n*=2~6, and they exhibited similar trends for different methylene chain lengths, with *n*=2 exhibiting slightly shorter values than *n*=3, 4, 5, and 6.

**Figure 19:**
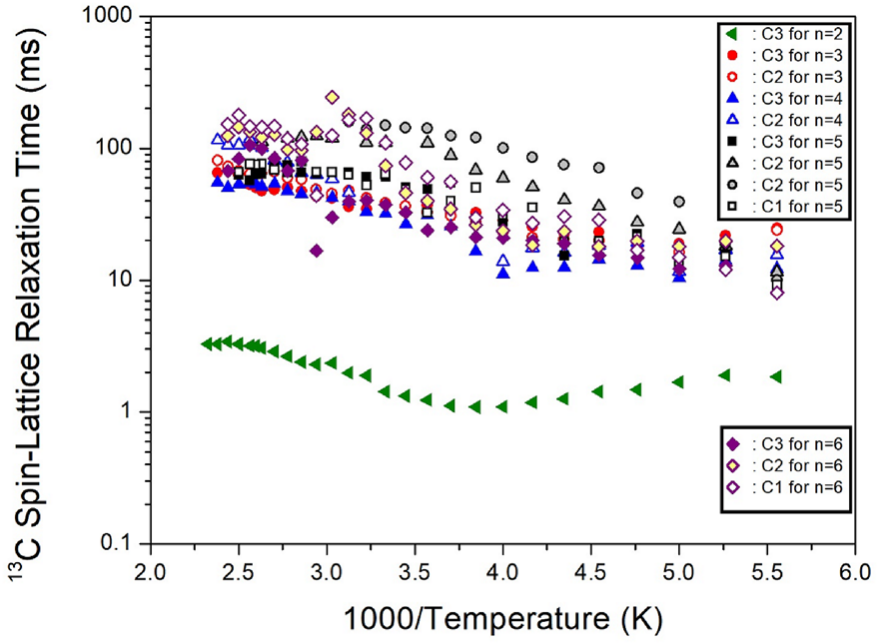
The ^13^C spin–lattice relaxation times in [NH_3_(CH_2_)_*n*_NH_3_]CuCl_4_ (*n* = 2, 3, 4, 5, and 6) as a function of inverse temperature^100–104^.

The ^13^C T_1ρ_ values for CH_2_-1, CH_2_-2, and CH_2_-3 in five crystals were obtained as a function of 1000/temperature from the slope of the logarithm of intensity versus the delay time plot. The decay curves for each carbon were represented by a single exponential function. When *n*=2, 3, 4, 5, and 6, by methylene length, the ^13^C T_1ρ_ at 300 K had values within the range of 1, 39, 32, 60-150, and 37-100 *ms*, respectively^100–104^. It is interesting to compare the results for ^13^C T_1ρ_ according to the alkyl chain lengths. The ^13^C T_1ρ_ values exhibited similar trends for *n*=3, 4, 5, and 6, with a very short value for *n*=2, as shown in Fig. 19. Unlike *n*=3, 4, 5, and 6, energy transfer was easier for the short alkyl chain length (*n*=2).

The ^1^H and ^13^C T_1ρ_ values exhibited a similar trend upon increasing the methylene chain length, with *n*=2 exhibiting shorter T_1ρ_ values than *n*=3, 4, 5, and 6. T_1ρ_ increased according to the length of the CH_2_ chain, indicating that energy transfer became more difficult. ^1^H T_1ρ_ values are short for [NH_3_(CH_2_)_*n*_NH_3_]CuCl_4_, including the paramagnetic ions. The paramagnetic Cu^2+^ ions bonded with the inorganic layer through N–H···Cl hydrogen bonds in [NH_3_(CH_2_)_*n*_NH_3_]CuCl_4_ directly affected the ^1^H environment.

### [NH_3_(CH_2_)_*n*_NH_3_]ZnCl_4_ (*n*=2, 3, 4, 5, and 6)

#### Crystal structures

**Figure 20:**
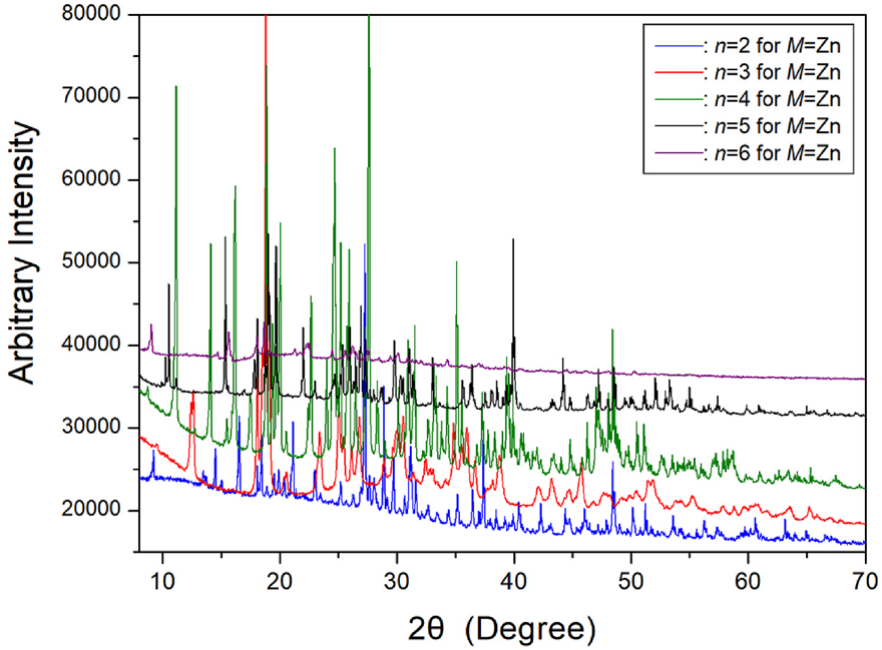
The powder X-ray diffraction patterns of [NH_3_(CH_2_)_*n*_NH_3_]ZnCl_4_ (*n* = 2, 3, 4, 5, and 6) at 300 K.^106,108,111^.

The SCXRD and PXRD experiments were conducted on [NH_3_(CH_2_)_*n*_NH_3_]ZnCl_4_ crystals, where *n* is 2, 3, 4, 5, and 6, at 300 K. The resulting PXRD patterns are presented in Fig. 20. Specifically, [NH_3_(CH_2_)_2_NH_3_]ZnCl_4_ with *n*=2 exhibits an orthorhombic structure with the *P2*_*1*_*2*_*1*_*2*_*1*_ space group. The unit cell parameters are *a*=8.832 Å, *b*=9.811 Å, *c*=11.089 Å, and Z=4^83^. For [NH_3_(CH_2_)_3_NH_3_]ZnCl_4_, the lattice constants are determined as *a*=10.670 Å, *b*=10.576 Å, *c*=10.755 Å, and *β*=118.477°, with the space group being *P2*_*1*_*/n*^81,106^. SCXRD experiments were carried out on [NH_3_(CH_2_)_4_NH_3_]ZnCl_4_, revealing a triclinic structure with the *P1*(bar) space group. The lattice parameters were found to be *a*=7.2839 (1) Å, *b*=8.1354 (1) Å, *c*=10.4592 (2) Å, and *α*=77.6527 (5)°, *β*=80.3358 (4)°, *γ*=82.8355 (5)°^107^. Moreover, [NH_3_(CH_2_)_5_NH_3_] ZnCl_4_ crystallized in a monoclinic structure with a *C2/c* space group and lattice constants *a*=21.4175 Å, *b*=7.3574 Å, *c*=19.1079 Å, *β*=120.5190°, and Z=8^108^. The SCXRD result of [NH_3_(CH_2_)_6_NH_3_]ZnCl_4_ revealed a triclinic system with the *P1*(bar) space group, and the cell constants were determined as *a*=7.2844 Å, *b*=10.1024 Å, *c*=10.1051 Å, *α*=74.3060°, *β*=85.9270°, *γ*=88.0170°, with Z=2^22,109^. In Fig. 1b, the structure of the organic [NH_3_(CH_2_)_6_NH_3_] cation and inorganic ZnCl_4_ anion in the [NH_3_(CH_2_)_6_NH_3_]ZnCl_4_ crystal is illustrated. In ZnCl_4_, the Zn atoms are surrounded by four Cl atoms, forming a tetrahedron. The (ZnCl_4_)^2−^ anion exists as an isolated tetrahedron, connected to the [NH_3_(CH_2_)_6_NH_3_] cation through N−H···Cl hydrogen bonds. Additionally, Table 4 provides information on the structures, space groups, and lattice constants of [NH_3_(CH_2_)_*n*_NH_3_]ZnCl_4_ (*n*=2, 3, 4, 5, and 6) crystals, revealing monoclinic structures for odd values of *n*, triclinic structures for even values of *n*, and orthorhombic structures for *n*=2.

**Table 4:** The structures, space groups, and lattice constants (Å) of [NH_3_(CH_2_)_*n*_NH_3_]ZnCl_4_ (*n* = 2, 3, 4, 5, and 6) crystals.

*n*	2	3	4	5	6
Structure	Orthorhombic	Monoclinic	Triclinic	Monoclinic	Triclinic
Space group	*P2*_*1*_*2*_*1*_*2*_*1*_	*P2*_*1*_*/n*	*P1*(bar)	*C2/c*	*P1*(bar)
Lattice constants					
a (Å)	8.832	10.670	7.2839	21.4175	7.2844
* b* (Å)	9.811	10.576	8.1354	7.3574	10.1024
* c* (Å)	11.089	10.755	10.4592	19.1079	10.1051
*α* (^∘^)			77.6527		74.3060
*β* (^∘^)		118.477	80.3358	120.5190	85.9270
γ (^∘^)			82.8355		88.0170
*Z*	4	4	2	8	2
References	^83^	^81,106^	^107^	^108^	^22,109^

#### Phase transition temperatures

**Figure 21:**
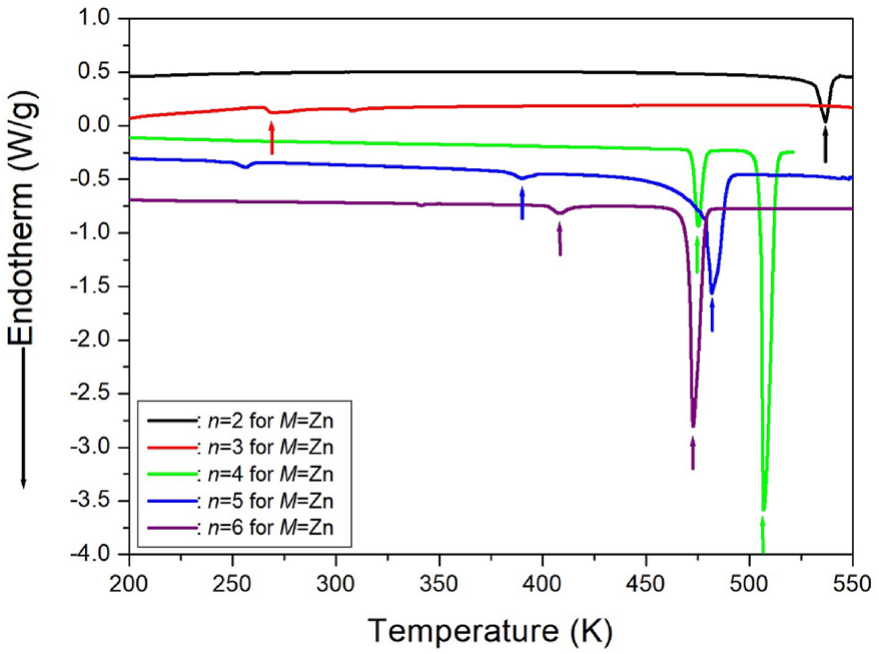
The differential scanning calorimetry curves of [NH_3_(CH_2_)_*n*_NH_3_]ZnCl_4_ (*n* = 2, 3, 4, 5, and 6) crystals^105,106,108,109^.

The DSC curves for [NH_3_(CH_2_)_*n*_NH_3_]ZnCl_4_ crystals, where *n* ranges from 2 to 6, are presented in Fig. 21. In the case of [NH_3_(CH_2_)_2_NH_3_]ZnCl_4_ (*n*=2), the DSC curve did not show any structural phase transition even above the decomposition temperature of 534 K^105^. For [NH_3_(CH_2_)_3_NH_3_]ZnCl_4_, only one endothermic peak at 268 K was observed, indicating a phase transition at this temperature^106^. In the case of [NH_3_(CH_2_)_4_NH_3_]ZnCl_4_ (*n*=4), two endothermic peaks at 481 and 506 K were identified on the DSC curve, suggesting a phase transition^106^. The DSC result for [NH_3_(CH_2_)_5_NH_3_]ZnCl_4_ indicated a very strong endothermic peak at 481 K, while two weaker peaks at 256 and 390 K were observed. The phase-transition temperatures obtained from the DSC results were compared with those from SCXRD and PXRD patterns. The small peak at 256 K in the DSC curve was determined to be unrelated to the phase transition. The phase-transition temperature was defined as T_C_=390 K, and the melting temperature was determined as T_m_=481 K^108^. In the case of *n*=6, the DSC thermogram revealed a weak endothermic peak at 408 K and a strong endothermic peak at 473 K. Further confirmation of the phase transition temperature at 408 K and the melting point at 473 K was obtained through PXRD and polarizing experiments, accounting for temperature changes^109^.

#### Thermodynamic properties

**Figure 22:**
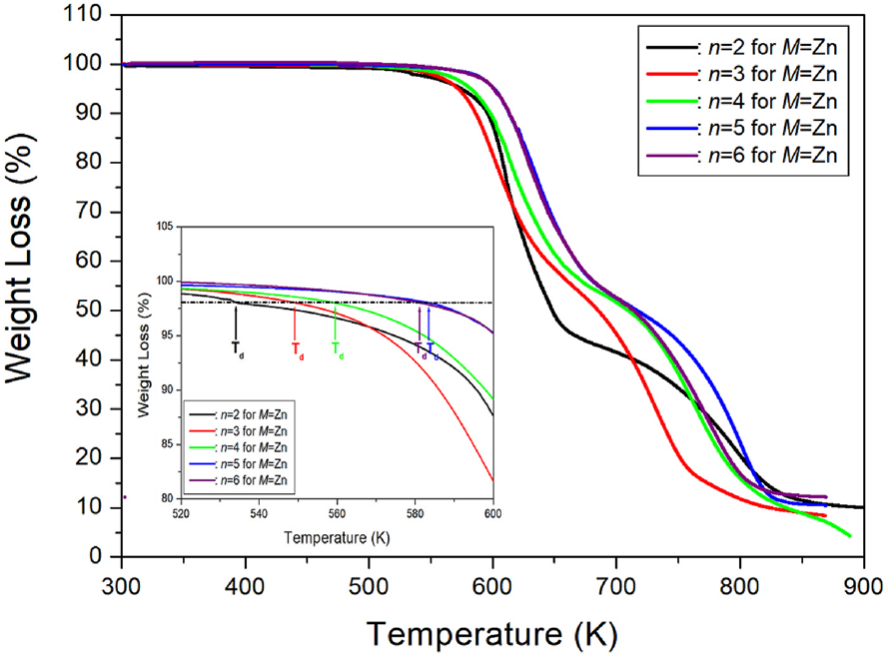
The thermogravimetry analysis curves of [NH_3_(CH_2_)_*n*_NH_3_]ZnCl_4_ (*n* = 2, 3, 4, 5, and 6)^105–109^.

TGA analysis was conducted on [NH_3_(CH_2_)_*n*_NH_3_]ZnCl_4_ crystals, where *n* ranges from 2 to 6, using the same DSC heating rate. The TGA results are depicted in Fig. 22, indicating that the crystals exhibit nearly stable behavior up to approximately 534, 549, 559, 583, and 581 K for *n* values of 2, 3, 4, 5, and 6, respectively^108,109^. For [NH_3_(CH_2_)_2_NH_3_]ZnCl_4_, above 534 K, a molecular weight loss is observed with increasing temperature. Weight losses of about 13 and 27 % occur near 589 and 618 K, respectively, attributed to the partial thermal decomposition of HCl and 2HCl moieties. TGA experiments with [NH_3_(CH_2_)_3_NH_3_]ZnCl_4_ reveal an onset of thermal decomposition (T_d_) at 549 K. In the case of *n*=4, the TGA result remains stable up to approximately 559 K. Weight losses of 12 and 25% due to the loss of HCl and 2HCl moieties occur at temperatures of 604 and 622 K, respectively, with a total weight loss of 95% near 900 K. For [NH_3_(CH_2_)_5_NH_3_]ZnCl_4_ crystals, molecular weight loss initiates at approximately 583 K, indicating partial thermal decomposition. Two-step decomposition processes are observed: first, a weight loss of 45% occurs at 685 K, and second, a weight loss of 95% occurs at 825 K. The 45% weight loss is attributed to organic decomposition, reaching 90% weight reduction, indicating almost complete decomposition of the organic component, leaving only Zn. Finally, the thermal characteristics of [NH_3_(CH_2_)_6_NH_3_]ZnCl_4_ crystals were assessed, and the DTA curve exhibited a peak around 473 K, consistent with the melting temperature determined from DSC and optical polarizing microscope analyses. The TGA and DTA curves indicate thermal stability up to about 581 K (T_d_). Weight loss rapidly decreases between 600 and 800 K, with approximately 22 % weight loss near 636 K due to the decomposition of 2HCl. Around 800 K, a 90 % weight loss occurs, resulting from the decomposition of NH_2_(CH_2_)_*n*_NH_2_·2HCl in all five compounds.

#### MAS NMR chemical shifts and spin-lattice relaxation times

**Figure 23:**
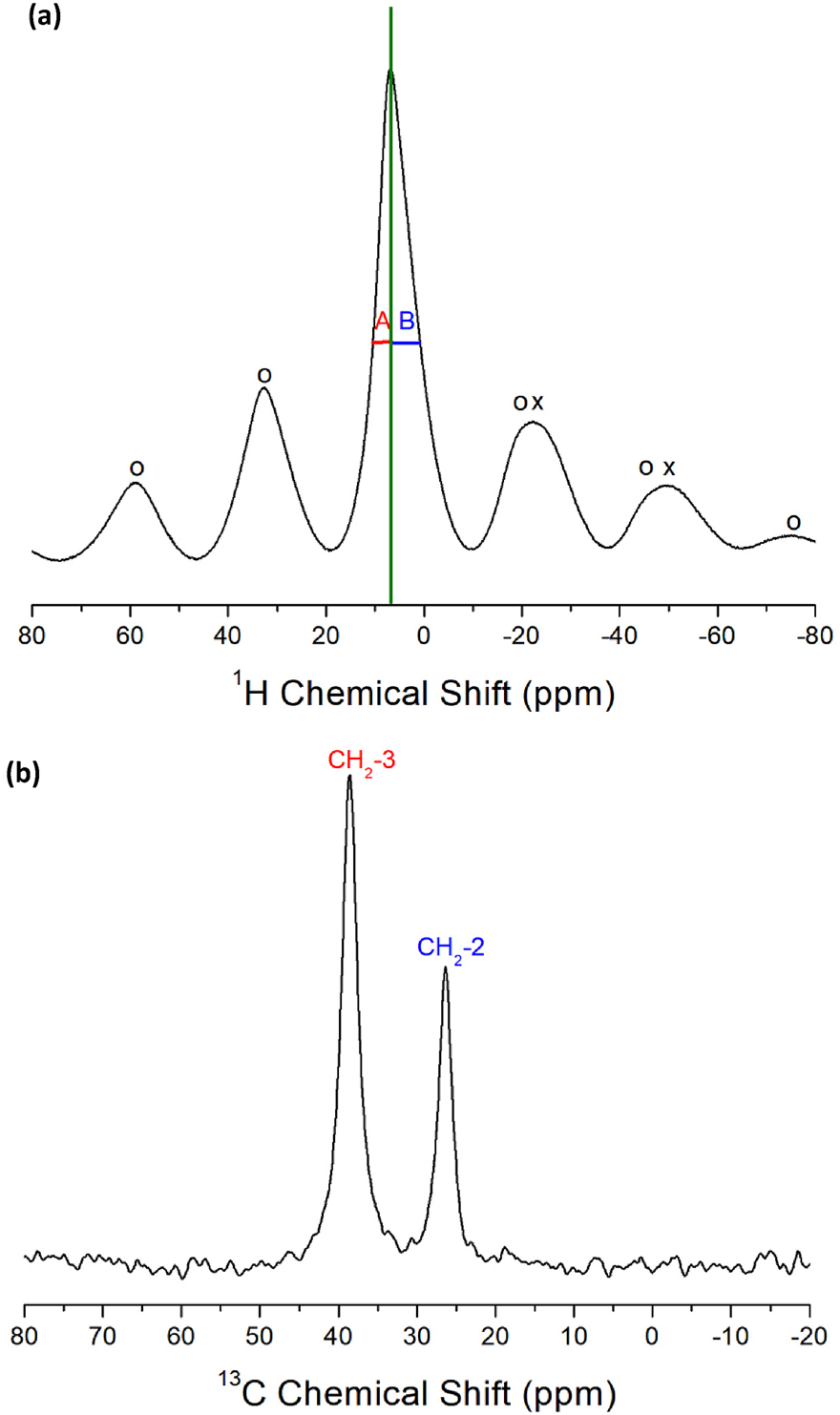
(**a**) The ^1^H NMR chemical shifts of [NH_3_(CH_2_)_3_NH_3_]ZnCl_4_ at 300 K. The open circles and crosses are sidebands for NH_3_ and CH_2_, respectively^106^. (**b**). The ^13^C NMR chemical shifts of [NH_3_(CH_2_)_3_NH_3_]ZnCl_4_ at 300 K^106^.

The ^1^H NMR chemical shifts of [NH_3_(CH_2_)_*n*_NH_3_]ZnCl_4_ crystals (*n*=2, 3, 4, 5, and 6) were recorded using MAS NMR spectroscopy. In the case of [NH_3_(CH_2_)_3_NH_3_]ZnCl_4_ with *n*=3, only one peak in the NMR spectra was observed, resulting from the overlap of NH_3_ and CH_2_ signals. The observed resonance signal was asymmetric, as illustrated in Fig. 23a. The line widths, represented as symbols A and B at the half-maximum value, differ from those at 3.54 and 6.11 ppm, respectively^106^. This asymmetry is attributed to the overlapping lines of the two ^1^H signals for CH_2_ and NH_3_ in the [NH_3_(CH_2_)_3_NH_3_] cations. At 300 K, the ^1^H NMR chemical shift was observed at δ=6.74 ppm. Spinning sidebands were marked with open circles and crosses.

In the case of *n*=3, MAS ^13^C NMR chemical shifts were measured with increasing temperature. Two resonance signals were observed in the MAS ^13^C NMR spectra of the compounds. The ^13^C chemical shifts for a spinning rate of 10 kHz were obtained, and the chemical shift for ^13^C was set as a standard reference for ^13^C in TMS. Specifically, the signals corresponding to the CH_2_-2 and CH_2_-3 carbon atoms in [NH_3_(CH_2_)_3_NH_3_]ZnCl_4_ at 300 K appeared at δ=26.41 and δ=38.53 ppm, respectively, as depicted in Fig. 23b.

**Figure 24:**
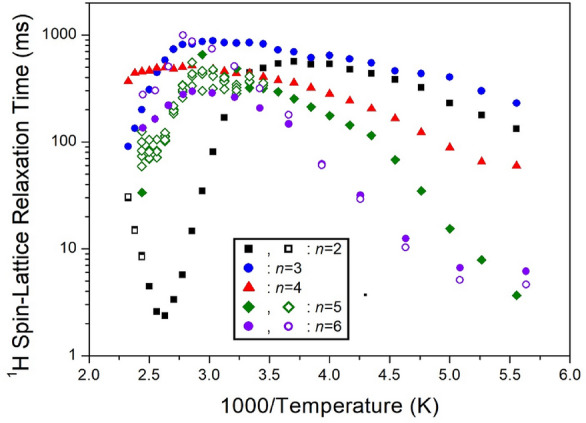
The ^1^H spin–lattice relaxation times in [NH_3_(CH_2_)_*n*_NH_3_]ZnCl_4_ (*n* = 2, 3, 4, 5, and 6) as a function of inverse temperature^105–109^.

The ^1^H MAS NMR spectra were measured with varying delay times, and the plot of spectral intensities vs. delay times was obtained using a single exponential function described by Eq. (1). The ^1^H T_1ρ_ values at 300 K were determined from the overlapping CH_2_ and NH_3_ peaks by analyzing the slope of the intensities vs. delay times results. The T_1ρ_ values for the ^1^H of NH_3_ and CH_2_ in [NH_3_(CH_2_)_*n*_NH_3_]ZnCl_4_ (*n*=2, 3, 4, 5, and 6) were obtained and are presented in Fig. 24 as a function of 1000/temperature. For methylene lengths *n*=2, 3, 4, 5, and 6, the ^1^H T_1ρ_ values at 300 K showed values of 444, 849, 440, 320~410, and 261~510 *ms*, respectively^105–109^. As the temperature increased, the ^1^H T_1ρ_ values also increased, and the values of T_1ρ_ above 300 K exhibited variations, as illustrated in Fig. 24. The most notable case was *n*=2, where T_1ρ_ gradually increased with rising temperature, reaching a maximum value at 270 K before rapidly decreasing above 300 K. Additionally, T_1ρ_ reached its minimum value near 380 K and then tended to increase again. This trend between 300 and 430 K indicates the presence of molecular motion. The T_1ρ_ values influenced by thermal motion are related to the correlation time (τ_C_) for molecular motion, according to the Bloembergen-Purcell-Pound theory^116^. Unlike the case of Mn, Co, and Cu containing paramagnetic ions, T_1ρ_ exhibited very long values in the case of Zn without paramagnetic ions.

**Figure 25:**
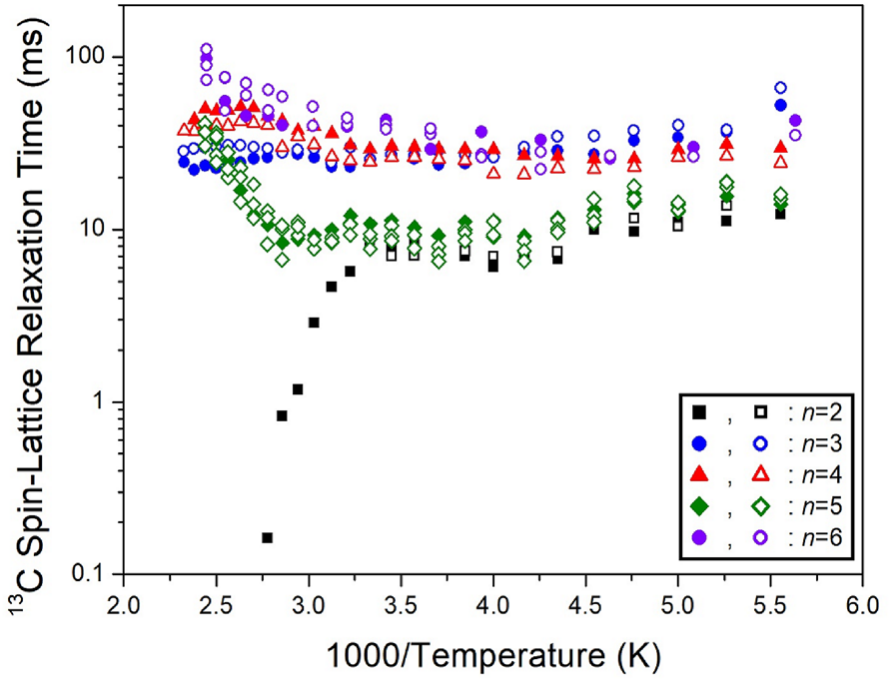
The ^13^C spin–lattice relaxation times in [NH_3_(CH_2_)_*n*_NH_3_]ZnCl_4_ (*n* = 2, 3, 4, 5, and 6) as a function of inverse temperature^105–109^.

The intensities of the ^13^C NMR signals were measured by varying the delay times, and the resulting ^13^C T_1ρ_ values for CH_2_-1, CH_2_-2, and CH_2_-3 in the cases of *n*=2, 3, 4, 5, and 6 were depicted in Fig. 25 as a function of 1000/temperature. These values exhibited similar trends for *n*=3, 4, and 6. However, the ^13^C T_1ρ_ values for *n*=2 displayed strong temperature dependence, sharply decreasing above 300 K with increasing temperature. Notably, there was a significant difference between the ^13^C T_1ρ_ values (10 times less) and the ^1^H T_1ρ_ values, indicating that energy transfer for ^13^C is more efficient. It is worth mentioning that when *n*=2, the ^1^H and ^13^C T_1ρ_ values exhibited different characteristics compared to *n*=3, 4, 5, and 6.

Furthermore, the length of the methylene did not distinctly affect the temperature dependence of ^1^H and ^13^C T_1ρ_. Instead, T_d_, the onset of thermal decomposition, showed temperature changes along the methylene length of the cation.

### [NH_3_(CH_2_)_*n*_NH_3_]CdCl_4_ (*n*=2, 3, 4, 5, and 6)

#### Crystal structures

**Figure 26:**
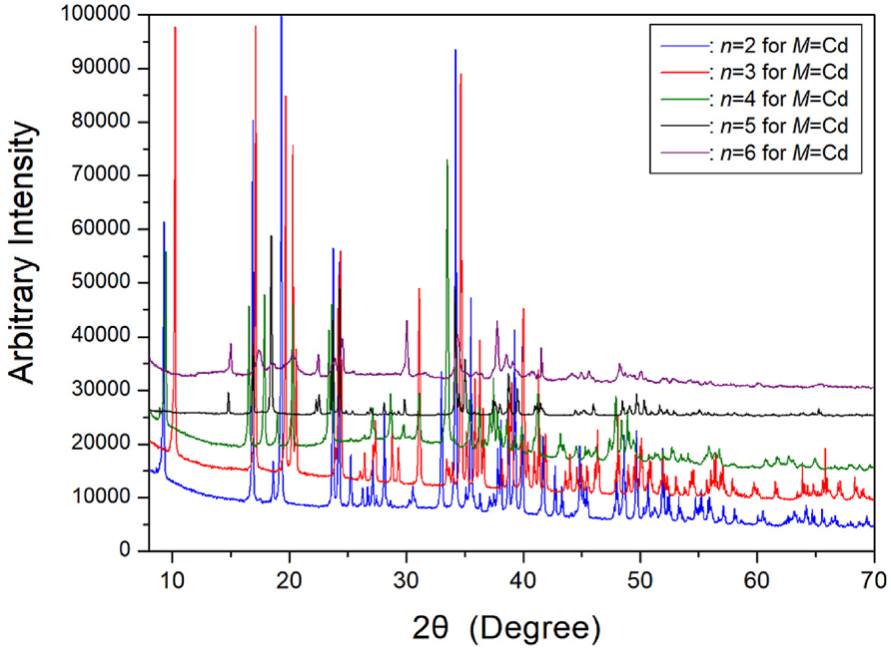
The powder X-ray diffraction patterns of [NH_3_(CH_2_)_*n*_NH_3_]CdCl_4_ (*n* = 2, 3, 4, 5, and 6) at 300 K^110,112,113^.

SCXRD and PXRD experiments were conducted on [NH_3_(CH_2_)_*n*_NH_3_]CdCl_4_ crystals (*n* = 2, 3, 4, 5, and 6) at room temperature, and their PXRD results are presented in Fig. 26. The space group and lattice constants for [NH_3_(CH_2_)_2_NH_3_]CdCl_4_ with *n*=2 were determined to be *P2*_*1*_*/a* and *a*=7.297 (2) Å, *b*=7.336 (2) Å, *c*=8.623 (2) Å, *β*=92.810 (11)°, Z=2^89,110^. For [NH_3_(CH_2_)_3_NH_3_]CdCl_4_ with *n*=3, the space group is *Pman*, and the lattice parameters are *a*=7.351 (6) Å, *b*=7.486 (5) Å, *c*=19.031 (14) Å, with Z=4^88,110^. In the case of [NH_3_(CH_2_)_4_NH_3_]CdCl_4_ with *n*=4, the space group is *P2*_*1*_*/a*, and the lattice parameters are *a*=7.663 (3) Å, *b*=7.593 (2) Å, *c*=9.514 (2) Å, *β*=101.616 (16)°, with Z=2^110^. The crystal structure of [NH_3_(CH_2_)_5_NH_3_]CdCl_4_ was determined to have a space group of *Pnam*, with lattice constants *a*=7.3292 (2) Å, *b*=7.5058 (2) Å, *c*=23.9376 (6) Å, and Z=4^87,112^. Finally, the structure of the [NH_3_(CH_2_)_6_NH_3_]CdCl_4_ crystal is monoclinic, with unit cell parameters *a*=7.305 Å, *b*=7.587 Å, *c*=23.997 Å, and *β*=91.22°^84,113^. The crystal structure of [NH_3_(CH_2_)_*n*_NH_3_]CdCl_4_ features slightly distorted CdCl_6_^2−^ ions, with the six hydrogen atoms in the terminal ammonium of the [NH_3_(CH_2_)_*n*_NH_3_] cation connected by N−H∙∙∙Cl interactions. The Cd atom is surrounded by six Cl atoms, forming nearly regular CdCl_6_ octahedra. The structural details, space groups, and lattice constants of [NH_3_(CH_2_)_*n*_NH_3_]CdCl_4_ (*n*=2, 3, 4, 5, and 6) crystals are summarized in Table 5. Notably, the structures are monoclinic when *n* is even and orthorhombic when *n* is odd.

**Table 5:** The structures, space groups, and lattice constants (Å) of [NH_3_(CH_2_)_*n*_NH_3_]CdCl_4_ (*n* = 2, 3, 4, 5, and 6) crystals.

*n*	2	3	4	5	6
Structure	Monoclinic	Orthorhombic	Monoclinic	Orthorhombic	Monoclinic
Space group	*P2*_*1*_*/a*	*Pman*	*P2*_*1*_*/a*	*Pman*	
Lattice constants					
a (Å)	7.297	7.351	7.663	7.3292	7.305
* b* (Å)	7.336	7.486	7.593	7.5058	7.587
* c* (Å)	8.623	19.031	9.514	23.9376	23.997
*β* (^∘^)	92.810		101.616		91.22
*Z*	2	4	2	4	
References	^89,110^	^88,110^	^110^	^87,112^	^84,113^

#### Phase transition temperatures

**Figure 27:**
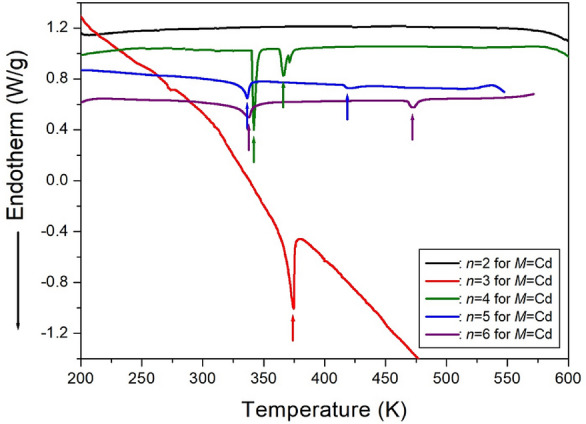
The differential scanning calorimetry curves of [NH_3_(CH_2_)_*n*_NH_3_]CdCl_4_ (*n* = 2, 3, 4, 5, and 6) crystals.^110–113^.

The DSC curves of [NH_3_(CH_2_)_*n*_NH_3_]CdCl_4_ (*n*=2, 3, 4, 5, and 6) are presented in Fig. 27. No peak was observed for *n*=2, while only one endothermic peak at 374 K was observed for *n*=3. In the case of *n*=4, two endothermic peaks were observed at 341 K and 366 K^110^. For the [NH_3_(CH_2_)_5_NH_3_]CdCl_4_ crystal, two endothermic peaks were observed at 336 K and 418 K^112^. The enthalpy for the phase transition is 3.17 kJ/mol at 336 K and 0.55 kJ/mol at 418 K, respectively. Finally, in the case of [NH_3_(CH_2_)_6_NH_3_]CdCl_4_, two endothermic peaks with enthalpies of 3.27 and 0.93 kJ/mol were observed at 337 K and 472 K, respectively^113^. These two peaks correspond to the phase transition temperatures.

#### Thermodynamic properties

**Figure 28:**
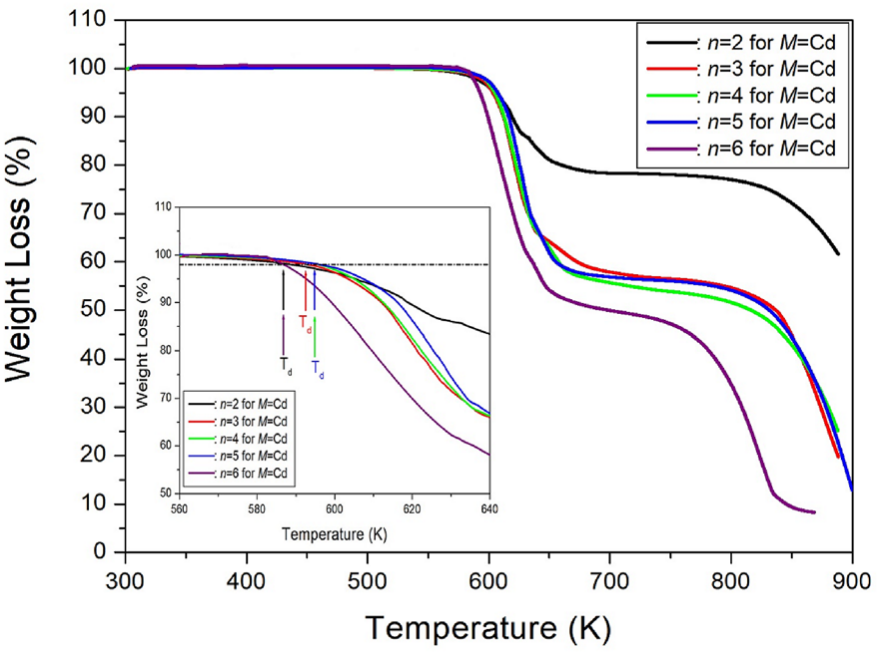
The thermogravimetry analysis curves of [NH_3_(CH_2_)_*n*_NH_3_]CdCl_4_ (*n* = 2, 3, 4, 5, and 6)^110–113^.

The TGA curves presented in Fig. 28 reveal that the [NH_3_(CH_2_)_*n*_NH_3_]CdCl_4_ crystals with *n*=2, 3, 4, 5, and 6 are nearly stable up to approximately 587, 592, 595, 595, and 587 K, respectively^110–113^, marking the onset of partial thermal decomposition corresponding to the number *n* of CH_2_ groups in the carbon chain. These compounds undergo breakdown near 650 K, experiencing a loss in molecular weight as the temperature rises. The remaining amount as solid residues can be calculated based on the molecular weights. In the case of *n*=2, a loss of 12 and 23% of its weight at temperatures of about 622 and 804 K was attributed to the partial decomposition of HCl and 2HCl, respectively. Similarly, weight losses of 11 and 22% occurred at temperatures of 613 and 623 K in the case of *n*=3. For [NH_3_(CH_2_)_4_NH_3_]CdCl_4_ with *n*=4, at temperatures of 612 and 623 K, 11 and 21% of their weight were lost, respectively. In the case of *n*=5, the 10 and 20% weight losses at temperatures of about 617 and 626 K were attributed to the partial thermal decomposition of HCl and 2HCl, respectively. Furthermore, weight loss at approximately 800 and 900 K was observed to be 46 and 87%, respectively. Finally, in the case of *n*=6, the weight loss rapidly decreased between 600 and 800 K, with a weight loss of about 20% occurring near 625 K due to the decomposition of 2HCl. Notably, when Cd is included, there is a significant difference in weight loss at high temperatures. Specifically, a loss of 25% in the case of *n*=2 occurs around 800 K, while *n*=3, 4, and 5 show a loss of 45%, and *n*=6 exhibits a loss of 65 %.

#### MAS NMR chemical shifts and spin-lattice relaxation times

**Figure 29:**
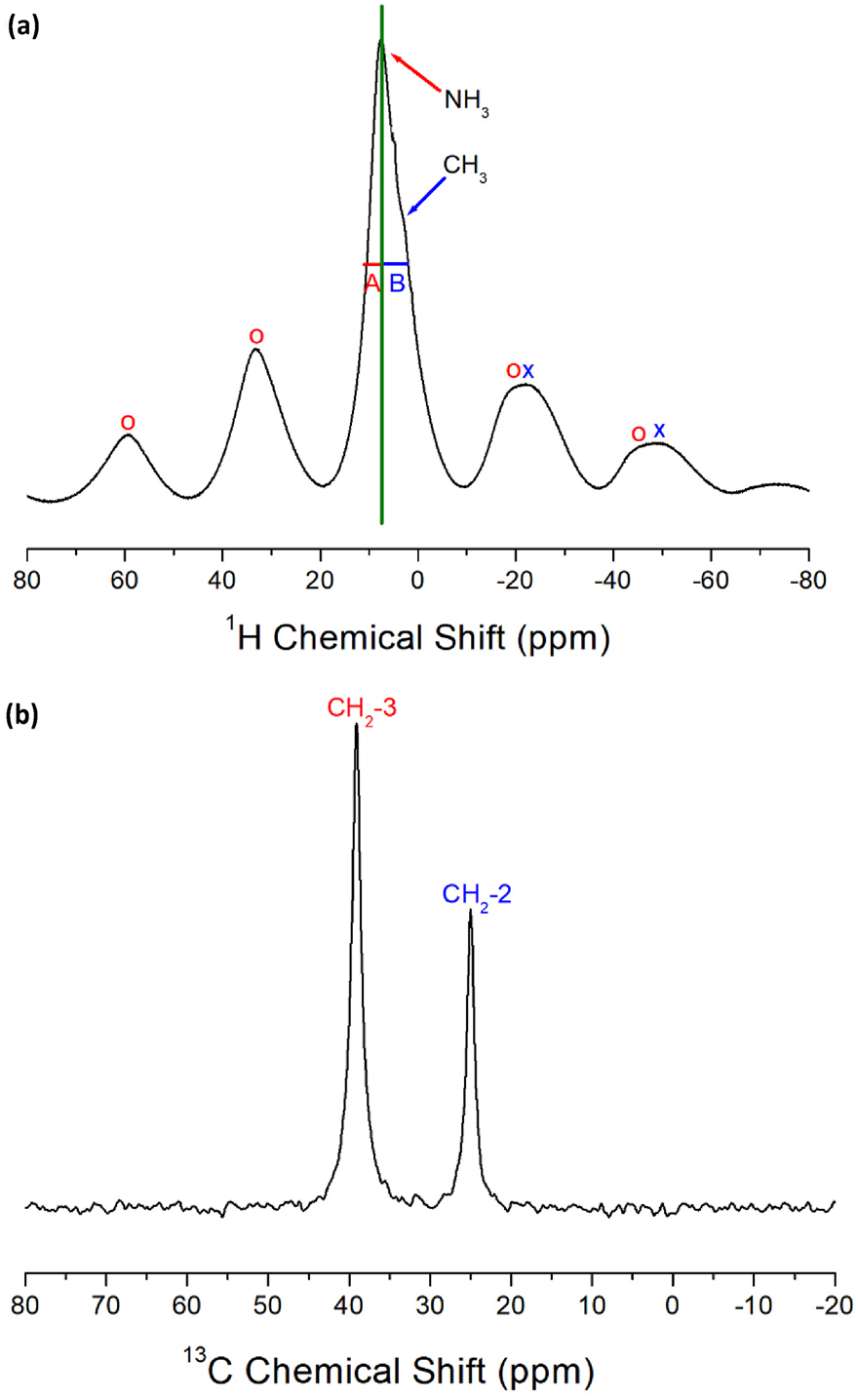
(**a**) The ^1^H NMR chemical shifts of [NH_3_(CH_2_)_3_NH_3_]CdCl_4_ at 300 K. The open circles and crosses are sidebands for NH_3_ and CH_2_, respectively^110^. (**b**) The ^13^C NMR chemical shifts of [NH_3_(CH_2_)_3_NH_3_]CdCl_4_ at 300 K^110^.

The MAS ^1^H NMR spectra of [NH_3_(CH_2_)_3_NH_3_]CdCl_4_ crystal with *n*=3 were recorded at 300 K, as shown in Fig. 29a. These resonance signals appeared asymmetric due to the overlapping of the ^1^H resonance lines of NH_3_ and CH_2_ in the [NH_3_(CH_2_)_3_NH_3_] cation. The spinning sidebands for NH_3_ and CH_2_ are denoted with open circles and crosses. At 300 K, the ^1^H chemical shift for CH_2_ was δ=3.23 ppm, whereas that for NH_3_ was δ=7.67 ppm^111^.

The ^13^C MAS NMR chemical shifts for CH_2_ in [NH_3_(CH_2_)_3_NH_3_]CdCl_4_ were recorded at different temperatures. At 300 K, the two resonance signals appeared at δ=25.04 and 39.07 ppm for CH_2_-2 and CH_2_-3, respectively, as shown in Fig. 29b. The ^13^C chemical shifts for CH_2_ were different for CH_2_-2, far away from NH_3_, and CH_2_-3, close to NH_3_. The line width of CH_2_-3 is wider than that of CH_2_-2.

**Figure 30:**
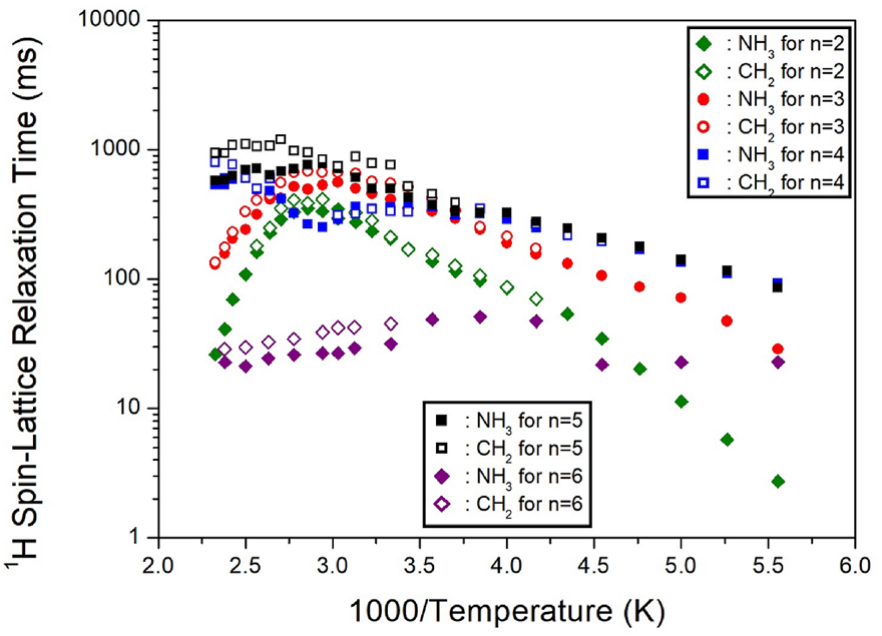
The ^1^H spin–lattice relaxation times in [NH_3_(CH_2_)_*n*_NH_3_]CdCl_4_ (*n* = 2, 3, 4, 5, and 6) as a function of inverse temperature^110–113^.

The ^1^H NMR spectra were measured with various delay times at each temperature for five crystals, and the slopes of the intensities vs. delay times followed a single exponential function. From the slope of the logarithm of intensities vs. delay times, the ^1^H T_1ρ_ values were obtained for NH_3_ and CH_2_. These values are shown in Fig. 30 for five crystals as a function of 1000/temperature. In the case of *n*=2, 3, 4, and 5, the ^1^H T_1ρ_ values increase rapidly as the temperature rises, and those of *n*=2 and 3 rapidly reduce at high temperatures. The ^1^H T_1ρ_ values of *n*=6 exhibited a slight dependence on temperature. It can be seen that ^1^H T_1ρ_ values according to the *n* values are different at high temperatures^110–113^.

**Figure 31:**
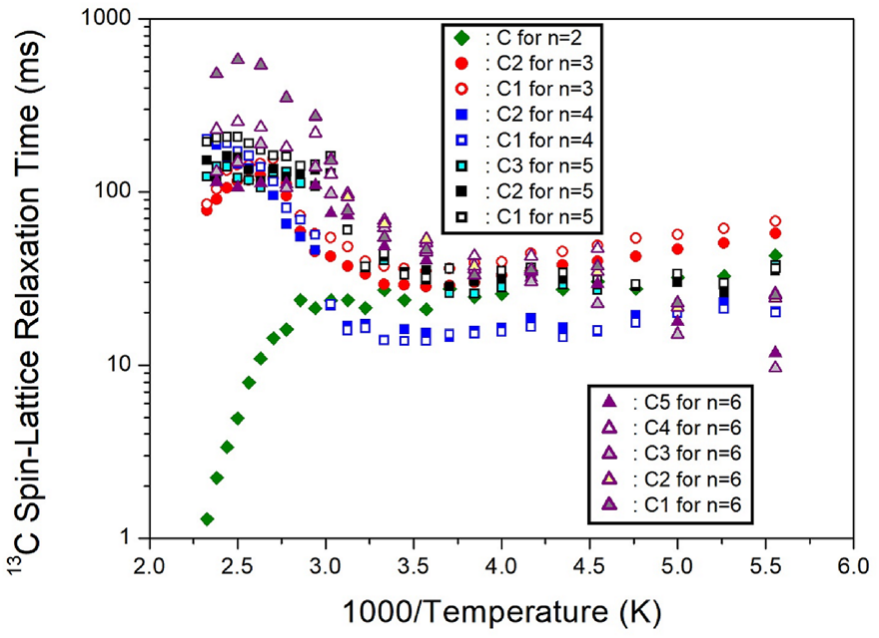
The ^13^C spin–lattice relaxation times in [NH_3_(CH_2_)_*n*_NH_3_]CdCl_4_ (*n* = 2, 3, 4, 5, and 6) as a function of inverse temperature^110–113^.

The intensities of the ^13^C NMR spectrum showed changes due to various delay times. The ^13^C T_1ρ_ values, obtained from the slope of their recovery traces, were determined for CH_2_-1, CH_2_-2, and CH_2_-3. Their results for five crystals are shown as a function of 1000/temperature in Fig. 31. In the case of *n*=2, ^13^C T_1ρ_ decreased slightly with a rise in temperature and decreased rapidly at high temperatures. For *n*=3, 4, 5, and 6, the ^13^C T_1ρ_ values decreased slightly with an increase in temperature, and increased again.

## Conclusion

In the pursuit of applications for an improved lead-free organic-inorganic perovskite-type solar cell, our research focused on identifying conditions conducive to organic-inorganic hybrid perovskite materials with high or no phase transition temperature, and high thermal stability. Consequently, we sought improved organic-inorganic hybrid perovskite materials by exploring the characteristics related to methylene length and the variation of various transition metals.

Single crystals of the organic-inorganic hybrid [NH_3_(CH_2_)_*n*_NH_3_]*M*Cl_4_ (*n*=2, 3, 4, 5, and 6; *M*=Mn, Co, Cu, Zn, and Cd) were grown using the aqueous solution method, and their crystal structures, phase transition temperatures (T_C_), and thermal decomposition temperatures (T_d_) were thoroughly examined. Additionally, our investigation delved into the impact of the even–odd number in the methylene length and various transition metals, holding potential implications for future applications.

From a structural standpoint, compounds involving Mn, Cu, and Cd, consisting of octahedral (*M*Cl_6_)^2−^ units, exhibited a monoclinic structure when *n* was even and an orthorhombic structure when *n* was odd. In contrast, for Co and Zn compounds with tetrahedral (*M*Cl_4_)^2−^ units, the crystal structure displayed an orthorhombic or triclinic arrangement when *n* was even and a monoclinic structure when *n* was odd.

Surprisingly, the phase transition temperatures did not exhibit a clear trend based on different transition metals (*M*=Mn, Co, Cu, Zn, and Cd), nor did they show a consistent pattern with varying methylene lengths of *n*=3, 4, 5, and 6. Remarkably, in the case of *n*=2, possessing the shortest methylene length, no phase transition temperature was observed. Notably, when the transition metal was Co, a relatively high phase transition temperature was recorded, as detailed in Table 6.

**Table 6:** The phase transition temperature by the methylene length of [NH_3_(CH_2_)_*n*_NH_3_]CdCl_4_ (*n* = 2, 3, 4, 5, and 6) crystals.

*n*	2	3	4	5	6
Mn	x	308, 338	378	298	
Co		483		494	
Cu	x	434	323	x	363
Zn	x	268	481, 506	256, 390	408
Cd	x	374	341, 366	336, 418	337, 472

**Figure 32:**
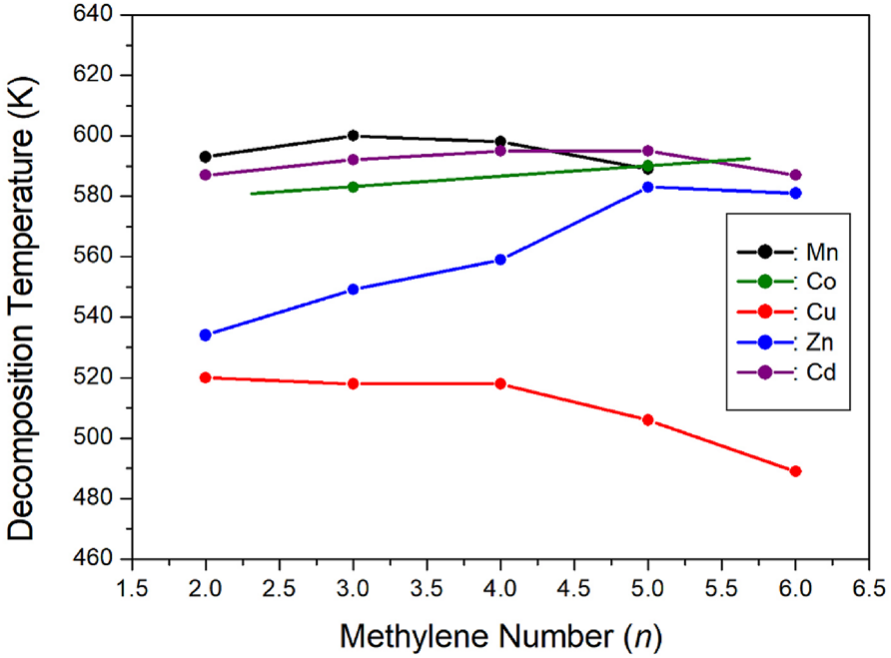
The relation between the decomposition temperatures and the methylene chain lengths of [NH_3_(CH_2_)_*n*_NH_3_]*M*Cl_4_ (*n* = 2, 3, 4, 5, and 6; *M* = Mn, Co, Cu, Zn, and Cd) crystals.

The results of comparative analysis and explanations for research on the thermal stabilities of [NH_3_(CH_2_)_*n*_NH_3_]*M*Cl_4_, where the methylene length varies as a parameter, are depicted in Fig. 32. In the case of transition metals Mn and Cd with an octahedral *M*Cl_6_^2-^ structure, the thermal decomposition temperature (T_d_) remains almost constant with respect to the methylene length, whereas for Co and Zn with a tetrahedral *M*Cl_4_^2-^ structure, T_d_ increases with the methylene length. Notably, in the case of Cu, T_d_ demonstrates a notable and rapid decrease. The changes in thermal decomposition temperature (T_d_) according to the transition metals can be explained by electronic configuration considerations. In the cases of Mn^2+^ and Co^2+^, where the 3*d* electron of the M shell is not filled and the valence electron is 4*s*^2^, as well as in the cases of Zn^2+^ and Cd^2+^, where the 3*d* electrons of the M shell and the 4*d* electrons of the N shell are filled, and the valence electrons are 4*s*^2^ and 5*s*^2^, respectively, T_d_ remains almost constant or increases as the methylene length increases. However, in the case of Cu^2+^, where the 3*d* electrons of the M shell are filled and the valence electrons are 4*s*^1^, T_d_ tends to decrease as the methylene length increases.

The NMR chemical shifts were found to be associated with the local field around the location of the resonance nucleus in the single crystals. Upon including metal ions (Mn, Co, Cu, Zn, and Cd) in [NH_3_(CH_2_)_*n*_NH_3_]*M*Cl_4_ (*n*=2, 3, 4, 5, and 6), the ^1^H and ^13^C chemical shifts exhibited the following trends: All ^1^H chemical shifts for the twenty-one compounds appeared at almost identical positions. However, the ^13^C chemical shifts displayed marked differences between the cases of paramagnetic ions (Mn, Co, and Cu) and the cases of Zn and Cd, which do not contain paramagnetic ions. Notably, ^1^H and ^13^C chemical shifts based on the methylene length *n* did not reveal any unusual patterns or trends.

The experiment results presented in Tables 7 and 8 highlight notable differences in ^1^H T_1ρ_ values based on the presence or absence of paramagnetic ions. When paramagnetic ions such as Mn, Co, and Cu are included, ^1^H T_1ρ_ exhibits very short values, whereas when Zn and Cd, which lack paramagnetic ions, are present, ^1^H T_1ρ_ values become considerably longer. Interestingly, ^13^C T_1ρ_ shows similar values regardless of the presence of paramagnetic ions. This suggests that ^1^H has a significant impact on T_1ρ_, while ^13^C has a minimal effect due to its distance from the paramagnetic ion. Examining T_1ρ_ values across different methylene lengths (*n*) reveals no significant differences or unusual patterns.

**Table 7:** The spin–lattice relaxation times T_1ρ_ (*ms*) for ^1^H of [NH_3_(CH_2_)_*n*_NH_3_]*M*Cl_4_ (*n* = 2, 3, 4, 5, and 6; *M* = Mn, Co, Cu, Zn, and Cd) crystals.

*M*	Mn	Co	Cu	Zn	Cd
2	20.8		7.79 ~ 8.95	449	204 ~ 212
3	15.4	0.016	12.98 ~ 14.84	844	411 ~ 551
4	14.4		12.91 ~ 14.29	411	334 ~ 357
5	0.0088 ~ 0.094	0.017	12.16 ~ 14.07	360	502 ~ 761
6	20.8		12.87 ~ 15.75	255	32 ~ 45

**Table 8:** The spin–lattice relaxation times T_1ρ_ (*ms*) for ^13^C of [NH_3_(CH_2_)_*n*_NH_3_]*M*Cl_4_ (*n* = 2, 3, 4, 5, and 6; *M* = Mn, Co, Cu, Zn, and Cd) crystals.

*M*	Mn	Co	Cu	Zn	Cd
2			1.41	8.18	27.67
3		0.025	38.11	18 ~ 21	29.13 ~ 37.13
4			32.36	24 ~ 29	13.90 ~ 14.06
5	1 ~ 19.14	0.038	60 ~ 150	7.7 ~ 10.9	40.11 ~ 43.83
6			37 ~ 112	39.53	48.40 ~ 54.55

Considering the overall trends of ^1^H and ^13^C T_1ρ_, *n*=3, 4, 5, and 6 exhibit almost similar trends as temperatures rise. In contrast, for *n*=2, a similar trend to *n*=3, 4, 5, and 6 is observed at low temperatures, but it undergoes a rapid shortening phenomenon at high temperatures. A longer T_1ρ_ implies increased difficulty in energy transfer from the nuclear spin to the surrounding environment. Consequently, the physicochemical properties of organic-inorganic hybrid perovskite [NH_3_(CH_2_)_*n*_NH_3_]*M*Cl_4_, influenced by various transition metal ions and the methylene length of the cation, hold potential applications as materials with lead-free, and high thermal stability attributes.

## Data Availability

The datasets generated and/or analysed during the current study are available from the corresponding author on reasonable request.
